# Identification of autosomal and sex chromosome aneuploidies using next generation sequencing

**DOI:** 10.1093/bioinformatics/btag104

**Published:** 2026-03-16

**Authors:** Nidia Barco-Armengol, Dèlia Yubero, Clara Xiol, Núria Catasús, Laura Martí-Sánchez, Judith Armstrong, Francesc Palau, Guerau Fernandez

**Affiliations:** Laboratory of Neurogenetics and Molecular Medicine, Center for Genomic Sciences in Medicine, Institut de Recerca Sant Joan de Déu, Esplugues de Llobregat, 08950, Spain; BarcelonaTech, Universitat Politècnica de Catalunya— (UPC), Barcelona, 08034, Spain; Genomics for the Diagnosis of Rare Diseases, Institut de Recerca Sant Joan de Déu, Esplugues de Llobregat, 08950, Spain; Department of Genetics, Hospital Sant Joan de Déu, Esplugues de Llobregat, 08950, Spain; Center for Biomedical Research Network on Rare Diseases (CIBERER), ISCIII, Madrid, 28029, Spain; Genomics for the Diagnosis of Rare Diseases, Institut de Recerca Sant Joan de Déu, Esplugues de Llobregat, 08950, Spain; Department of Genetics, Hospital Sant Joan de Déu, Esplugues de Llobregat, 08950, Spain; Department of Genetics, Hospital Sant Joan de Déu, Esplugues de Llobregat, 08950, Spain; Department of Genetics, Hospital Sant Joan de Déu, Esplugues de Llobregat, 08950, Spain; Genomics for the Diagnosis of Rare Diseases, Institut de Recerca Sant Joan de Déu, Esplugues de Llobregat, 08950, Spain; Department of Genetics, Hospital Sant Joan de Déu, Esplugues de Llobregat, 08950, Spain; Center for Biomedical Research Network on Rare Diseases (CIBERER), ISCIII, Madrid, 28029, Spain; Laboratory of Neurogenetics and Molecular Medicine, Center for Genomic Sciences in Medicine, Institut de Recerca Sant Joan de Déu, Esplugues de Llobregat, 08950, Spain; Center for Biomedical Research Network on Rare Diseases (CIBERER), ISCIII, Madrid, 28029, Spain; Division of Pediatrics, Faculty of Medicine and Health Sciences, University of Barcelona, Barcelona, 08007, Spain; Laboratory of Neurogenetics and Molecular Medicine, Center for Genomic Sciences in Medicine, Institut de Recerca Sant Joan de Déu, Esplugues de Llobregat, 08950, Spain; Department of Genetics, Hospital Sant Joan de Déu, Esplugues de Llobregat, 08950, Spain; Center for Biomedical Research Network on Rare Diseases (CIBERER), ISCIII, Madrid, 28029, Spain

## Abstract

**Motivation:**

Chromosomal abnormalities, referred to as aneuploidies, occur in approximately 0.3% of live births. While the majority of aneuploidies in humans are incompatible with life, well-characterized exceptions include Down syndrome (47,+21), Patau syndrome (47,+13), Edwards syndrome (47,+18), Turner syndrome (45,X0), Klinefelter syndrome (47,XXY), and triple X syndrome (47,XXX). These chromosomal alterations disrupt gene expression and cellular function, leading to genetic and developmental disorders. With the increasing adoption of next generation sequencing (NGS) in clinical diagnostics, this study aims to explore the potential use of NGS for aneuploidies detection.

**Results:**

Using data derived from clinical exomes (CES) and whole exomes (WES) sequencing we have been able to detect autosomal as well as sex chromosome aneuploidies with high specificity. Moreover, we have also been able to identify mosaic aneuploidies proving the high sensibility of this methodological approach. Thus, we present NGS as a cost-effective first line approach to detect chromosomal aneuploidies in routine diagnostic practice.

**Availability and implementation:**

Scripts are available at https://github.com/B-R-I-D-G-E/AneuploidiesStudies.

## 1 Introduction

Chromosomal segregation is the process through which the two sister chromatids formed during replication separate and migrate to opposite poles of the nucleus during cell division. Errors in the segregation process may arise as a result of structural issues in the microtubular spindle during cell division, that can finally lead to aneuploidies, structural alterations in the number of chromosomes within a cell (O’Connor 2008a, Compton 2011). If this chromosomal unbalanced segregation occurs during meiosis, it could affect the gamete and, consequently, the future embryo, leading to a chromosomal monosomy or trisomy. Meanwhile, if this error occurs during embryonic development, it could lead to mosaic aneuploidies, just affecting a subset of cells.

In most cases, aneuploidies are incompatible with life, especially when they occur on autosomes. The imbalance in gene dosage cause the disruption of essential cellular processes and leads to severe developmental defects. Moreover, monosomies also allow the influence of detrimental heterozygous mutations to manifest in hemizygosis, acting in a dominant manner. When viable, aneuplidies can result in significant developmental abnormalities. The three well-known examples of complete autosomal aneuploidies are Down syndrome, Patau syndrome, and Edwards syndrome. Down syndrome (Antonarakis *et al.* 2020) (one in 1000 newborns) (47,+21), is caused by trisomy of chromosome 21 resulting in alterations in the musculoskeletal, neurological, and cardiovascular systems. Down syndrome, is commonly associated with short stature, muscle hypotonia, atlantoaxial instability, reduced neuronal density, cerebellar hypoplasia, intellectual disability, and congenital heart defects. Patau syndrome (Williams and Brady 2023) (one in 5000 newborns) (47,+13) is caused by trisomy of chromosome 13. Individuals with this condition typically exhibit characteristic facial features such as a sloping forehead, small malformed ears, anophthalmia or microphthalmia, micrognathia, and preauricular appendages. Central nervous system anomalies, with alobar holoprosencephaly being the most common defect, are also present, as well as heart defects such as ventricular septal defect, atrial septal defect, tetralogy of Fallot, atrioventricular septal defect, and double-outlet right ventricle. Edwards syndrome (Balasundaram and Avulakunta 2024) (one in 5000 newborns) (47,+18) is caused by trisomy of chromosome 18, and may manifest a variety of neurological, craniofacial, skeletal, cardiovascular, pulmonary, gastrointestinal, genitourinary, and central nervous system malformations. These include psychomotor delay, intellectual disability, microcephaly, a triangular face, severe growth retardation, ventricular or atrial septal defects, patent ductus arteriosus, tetralogy of Fallot, pulmonary hypoplasia, pyloric stenosis, and hydrocephalus, among others.

In contrast to autosomal aneuploidies, sex chromosome aneuploidies typically exhibit less severe phenotipic consequences and frequently with no pathognomonic physical characteristics. As a result, they are often uncovered in adulthood, often due to fertility issues. Examples of sexual aneuploidies are: Turner syndrome, Klinefelter syndrome, and Trisomy X syndrome.

One of the most widely recognized sex chromosome aneuploidies affecting females is Turner syndrome (Shankar and Nagalli 2025) (one in 2000–2500 newborns) (X0). Women with this syndrome typically present short stature, premature ovarian failure, a low hairline at the base of the neck among others, and, in some cases, they may also have renal abnormalities, congenital heart defects, or developmental delay. Another well-studied sex chromosome aneuploidy is Klinefelter syndrome (https://rarediseases.info.nih.gov/espanol/13471/sindrome-de-klinefelter, https://www.genome.gov/Genetic-Disorders/Klinefelter-Syndrome) (one in 500–1000 newborns) that affects males and is characterized by disomy of the X chromosome (XXY). This disorder may be asymptomatic in some cases, while in others can lead to cognitive, social, or learning impairments. This condition is typically associated with hypogonadism, small testicles, gynecomastia, tall stature, and infertility. Genitourinary anomalies, such as hypospadias and micropenis, may also occur. Variants of Klinefelter syndrome that involve the presence of more than one additional X chromosome (such as XXXY or XXXXY) result in more severe phenotypic consequences, including intellectual disability, skeletal anomalies, and impaired coordination. Finally, Trisomy X syndrome (https://www.ncbi.nlm.nih.gov/medgen/113140, Otteret al. 2010) (one in 1000 newborns), is a disorder that affects females and is characterized by the presence of an additional X chromosome (XXX). Women with this condition may present above average heights, but generally exhibit no unusual physical characteristics. The majority exhibit normal sexual development and with no fertility issues, despite being associated with a higher likelihood of learning disabilities and delays in speech and language development. Patients with Trisomy X syndrome may also experience delay in motor skill development and muscle tone, as well as emotional and behavioral difficulties.

The presence of aneuploidies increases the risk of miscarriage and the birth of children with chromosomal abnormalities, especially in women of advanced maternal age.

Aneuploidies are currently detected using techniques such as karyotyping (O’Connor 2008b), FISH (O’Connor 2008c), aCGH (Theisen 2008), or QF-PCR (Upadhyay *et al.* 2024). These specific techniques are typically requested based on a clear clinical suspicion. However, because many aneuploidies result in variable and often subclinical phenotypes, their detection can be challenging, especially when targeted genetic tests are not routinely ordered.

In recent years, next generation sequencing (NGS) technology has been incorporated into the clinical practice for the diagnosis of suspected genetic disorders. NGS has proven to be a highly effective tool for rare Mendelian disorder diagnosis providing high accuracy in identifying nucleotide-level variants (Abolhassani *et al.* 2024). Although NGS has not been specifically designed for the detection of aneuploidies, in this study, we propose a method for detecting aneuploidies based on the normalized mean coverage value of different chromosomes. The comparison with a control population enables the identification of patients with autosomal or sex chromosome aneuploidies. Incorporating this approach into routine diagnostics allows the simultaneous detection of both NGS-based variants and aneuploidies in a single assay. This broadens the spectrum of detectable genetic alterations, improving the identification of clinically relevant or incidental findings that may benefit the patient.

## 2 Materials and methods

### 2.1 Samples

This study performed unsupervised analyses in 6611 samples from Sant Joan de Déu Children’s Hospital (HSJD) for the detection of aneuploidies in autosomes and sex chromosomes using coverage control datasets generated from 2603 non-tumoral mostly pediatric (<16 years) samples. The control dataset samples included 1594 clinical exomes (CES) capture CCP17 (Agilent Technologies Inc., Santa Clara, CA, USA) and 1009 whole exomes (WES) capture SSv8NCV (Agilent Technologies Inc., Santa Clara, CA, USA). WES samples were distributed in 780 dual-index samples and 229 single-index samples. Differential statistical significance was detected due to capture type and single/dual index (*P*-value <.01). Thus, analyses were grouped by capture and indexing method to avoid potential biases.

### 2.2 Bioinformatics pipeline

Coverage data for each sample was carried out through an in-house pipeline with multiple steps. Briefly, adapters removal and low-quality base trimming was performed using cutadapt (Martin 2011), applying a quality filter with a threshold of 20 and trimming low-quality bases with a threshold of 25. Next, the trimmed reads were aligned to the hg19 human reference genome using BWA-mem (Li and Durbin 2009). Duplicate and low mapping quality reads (MAPQ < 15) were removed using samtools (Danecek *et al.* 2021) rmdup and only on-target reads filtered with intersectBed (Quinlan and Hall 2010) were used for the coverage analysis. Finally, samtools using the depth function provided the coverage values for each base in the regions of interest, from which the mean coverage values for each chromosome were calculated.

### 2.3 Control datasets

An initial quality filter excluding samples with a mean coverage of autosomes lower than 50 was applied (Fig. S1A–C and Table S1, available as supplementary data at *Bioinformatics* online). The optimal coverage range begins at the minimum read depth (50×) where background noise is sufficiently reduced to ensure signal is separated by at least three standard deviations (SDs) from the normal baseline (*Z*-score > 3), guaranteeing statistical distinction with 99.9% confidence (Table S2, available as supplementary data at *Bioinformatics* online). Also, samples derived from tumor origin were excluded from the study, as these samples can lead to false positive aneuploidies due to its intrinsic chromosomal instability. Capture method (CES/WES) and indexing method (single/dual) were analysed independently. Kruskal–Wallis test on each normalized autosome by mean autosomes coverage values was used to determine if the variability assigned to capture method or indexing method was statistically significant (Table S3, available as supplementary data at *Bioinformatics* online).

The robustness of this methodology is further supported by the composition of our control dataset, which was generated from more than 400 independent pools collected over a seven-year period. Consequently, the results demonstrate a high degree of stability and reproducibility across independent batches. In order to identify potential deviations from the original cohort, it is recommended to conduct a comparative analysis when the method is implemented under new technical conditions. This is because the indexing method and the type of capture have present significant differences in this study.

Circos plots (Gu *et al.* 2014) and the KaryoploteR package (Gel and Serra 2017), were used for a detailed inspection of chromosomes coverage and its distribution. Noteworthy, WES and CES targeted regions comparison at the Y chromosome indicated that the WES analysis comprised a much wider region, with greater coverage, being significantly relevant specially when detecting sex aneuploidies (Fig. S2, available as supplementary data at *Bioinformatics* online).

### 2.4 Main analysis

Unsupervised analyses of all samples were conducted with the control dataset cohort in order to detect complete or mosaic aneuploidies. Samples with a mean coverage of autosomes lower than 50 were excluded from the analysis as in the control dataset (Fig. S1, available as supplementary data at *Bioinformatics* online). For autosomes, the mean coverage values for each chromosome were normalized by the mean coverage values of the other autosomes or the combined mean coverage of all remaining autosomes from the same sample. In order to optimize normalization, the SD for each autosome was calculated for each type of normalization, and the three best normalizations were selected (Table S4, available as supplementary data at *Bioinformatics* online). Chromosomes 13, 18, and 21 were excluded from the top three normalization due to the possibility of viable aneuploidies on these chromosomes (Figs. S3–S5, available as supplementary data at *Bioinformatics* online). These normalizations were subsequently used to conduct principal component analysis (PCA) using prcomp function from stats package in R (https://www.R-project.org/).

In order to analyse sex chromosome aneuploidies, the mean coverage of the X and Y chromosomes were analysed simultaneously, as their coverage is intrinsically linked to the sample’s karyotype. The mean coverage of sexual chromosomes was normalized using the mean coverage of autosomes and the combined mean coverage of all autosomes. The normalized values were plotted in scatter plots to facilitate the identification of outliers that might indicate aneuploidies. To evaluate whether there were significant differences between the outliers and the reference clusters (XX and XY), a distance matrix was calculated for the normalized values of the X and Y chromosomes, and the ADONIS2 test (Anderson 2001) was used. To optimize normalization for the sex chromosomes, beta-dispersions (Anderson 2006) of the main clusters were calculated.

### 2.5 Synthetic data

In order to validate autosomal aneuploidies, synthetic samples were generated with varying percentages of coverage, simulating mosaic aneuploidies ranging from 10% to 100%. Synthetic data were produced by subsampling a random individual from the control dataset. PCA analyses were conducted using the three best normalizations chosen in the previous unsupervised analysis of autosomes, with the SD recalculated to select the best normalizations with a control dataset. The interquartile range (IQR) was calculated with the PC1 values, to determine the threshold for mosaic aneuploidy at which a clear difference could be detected relative to the main sample cluster (PC1 > Q3 + 5.5 * IQR | PC1 < Q1 − 5.5 * IQR).

### 2.6 Outliers validation

The various outliers identified in the unsupervised analyses were subsequently validated using several reference orthogonal techniques commonly employed for the detection of aneuploidies, such as aCGH, karyotyping, FISH, or QF-PCR, in order to confirm the presence of the alteration.

## 3 Results

The normalization approach used in this study reduces variability among samples that may result from differences in sequencing depth or other technical factors, thereby enhancing the accuracy of aneuploidy detection and enabling more appropriate comparisons between samples.

### 3.1 Autosome aneuploidies study

**Figure 1:**
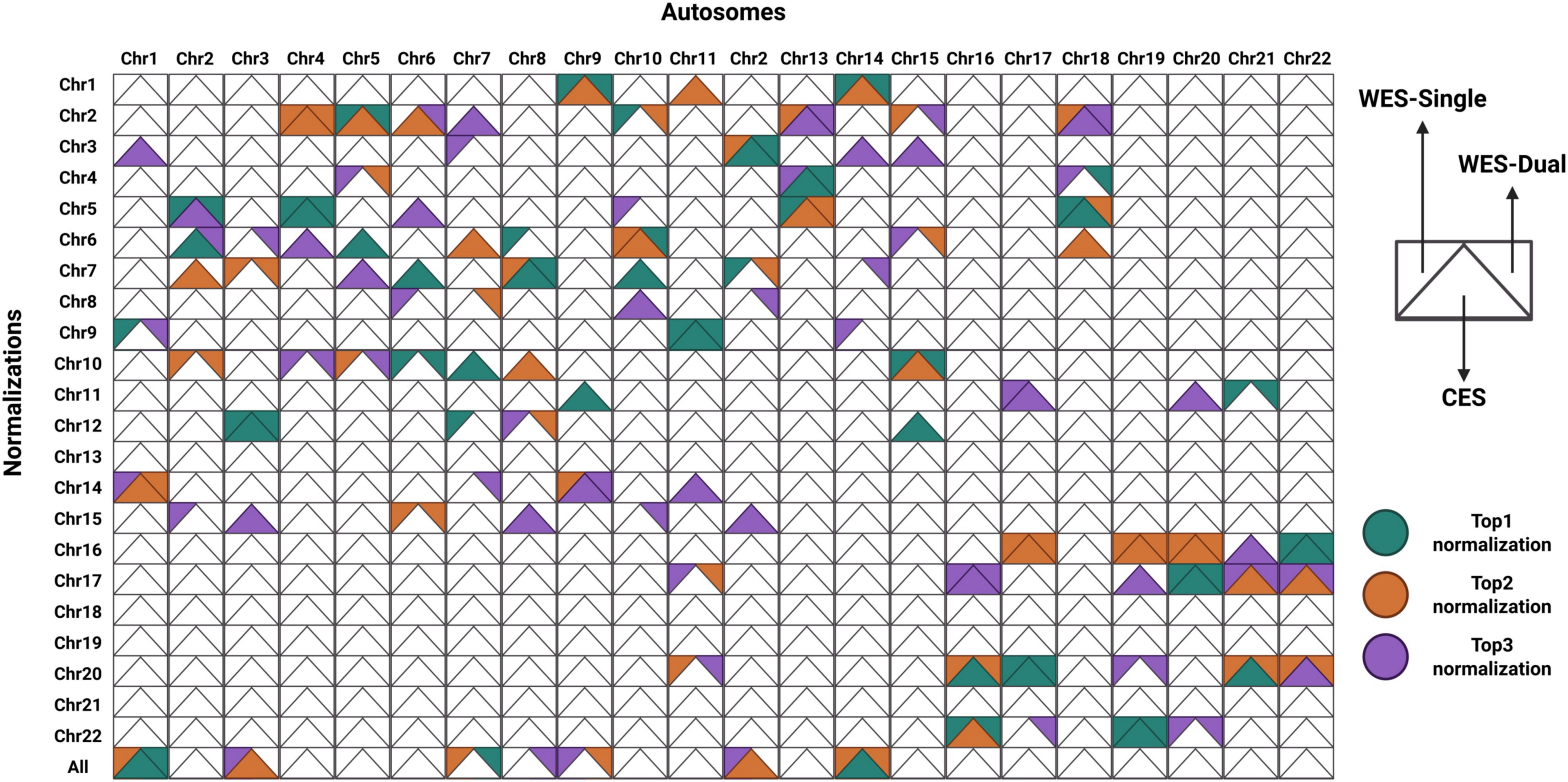
Autosomal three best normalizations. Top 1 normalization (green), Top 2 normalization (orange), and Top3 normalization (violet) and capture type CES (center), WES-Single (left), and WES-Dual (right) per chromosome are indicated.

For each autosome, the SD of the mean coverage values was calculated using the different chromosome normalizations. Top three normalizations with lower SD for each autosome were selected for aneuploidy detection (Fig. 1). As an example, when normalizing Chr1 coverage, the best normalization for CES data was with all autosomes except Chr1 (Top1), only using Chr14 (Top 2) and only using Chr3 (Top 3). For WES-Single data, the optimal normalizations were using Chr9, with all autosomes except Chr1, and using Chr14. And in the case of WES-Dual data, the best normalizations were with all autosomes except Chr1, using Chr14, and using Chr9. Similarly, for the remaining autosomes, the three best normalizations were identified.

Top three normalized mean coverages were visualized using PCA plots per autosomes as shown in Figs. S3–S5, available as supplementary data at *Bioinformatics* online.

In order to stablish an accurate threshold for considering a sample as a true outlier using PCA plots, synthetic samples were used. Simulated trisomies with different percentages of mosaicism in blue, and monosomies with different percentages of mosaicism in red were generated and compared to the control dataset (Figs. S3–S5, available as supplementary data at *Bioinformatics* online). PC1 values were used to determine if one sample was a clear outlier or not using the IQR.

**Figure 2:**
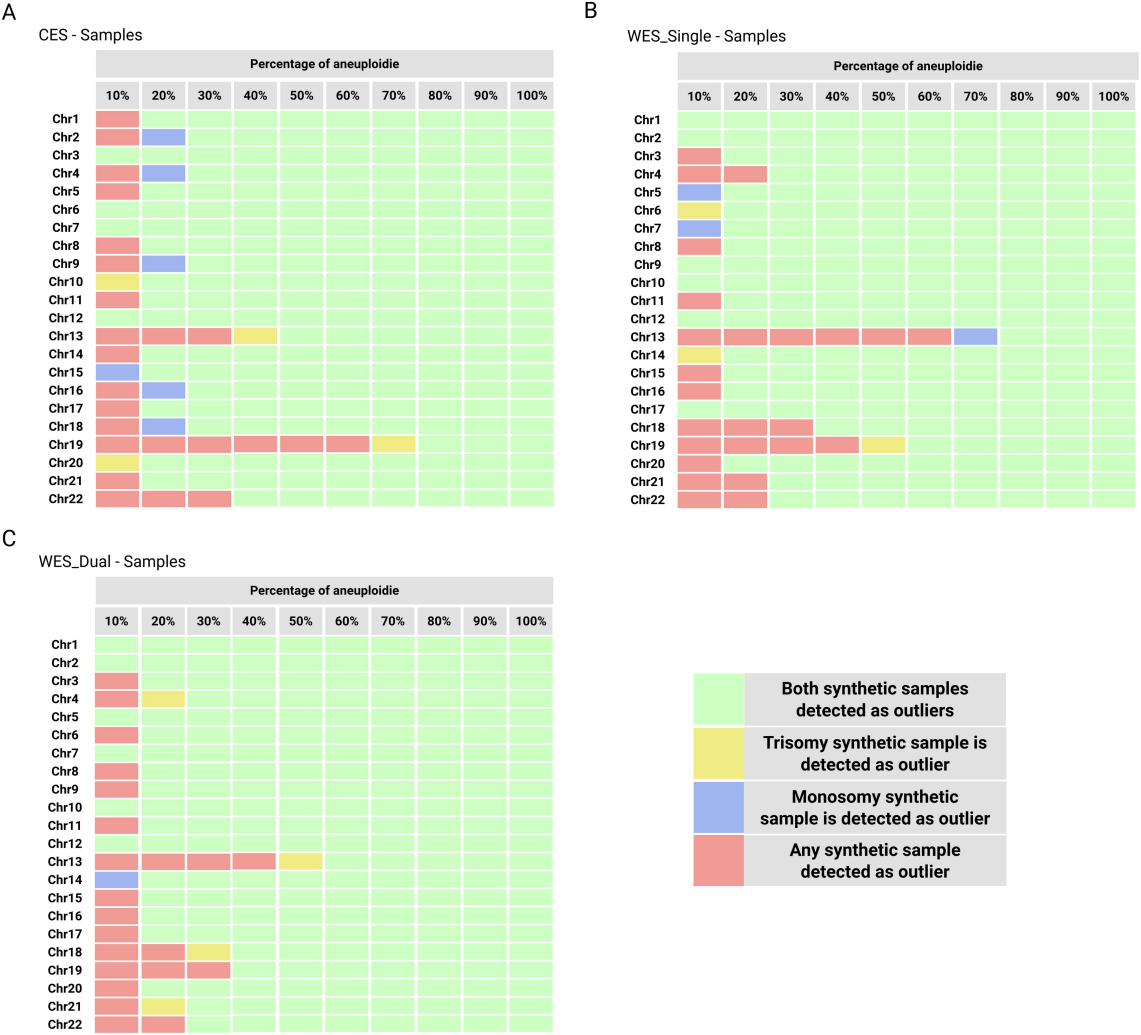
Determination of chromosomal aneuploidy thresholds using the control cohort. (A) CES, (B) WES-SingleIndex, (C) WES-DualIndex. Significant aneuploidies using synthetic mosaicism percentage that has allowed the identification of both trisomy as well as monosomy samples in green, absence of neither significant trisomy nor monosomy samples in red, identification of only trisomy samples in yellow, and identification of only monosomy samples in blue.

The evaluation of synthetic samples showed that this approach can reliably identify aneuploidies greater than 20% in most of autosomes (Fig. 2). Forty-four out of 66 autosomes (three different captures) showed significant mosaicism at 20% synthetic sample with respect to the control cohort allowing the identification of both trisomy and monosomy aneuploidies (marked in green). Some autosomes had skewed detection for gain or loss of aneuploidies. Eleven autosomes showed significant trisomies before monosomies (yellow) and 10 autosomes showed significant monosomies before trisomies (blue). No chomosome had more than 10% difference between detection of trisomy and monosomy. Nonetheless, certain autosomes, such as Chr13 or Chr19 aneuploidies can only be distinguished from controls at high percentages of mosaicism for all capture types. For Chr13, the threshold was determine at 50%, 80%, and 60% for CES, WES-Single, and WES-Dual, respectively. Meanwhile, for Chr19, threshold was determine at 80%, 60%, and 40% for CES, WES-Single, and WES-Dual, respectively.

**Figure 3:**
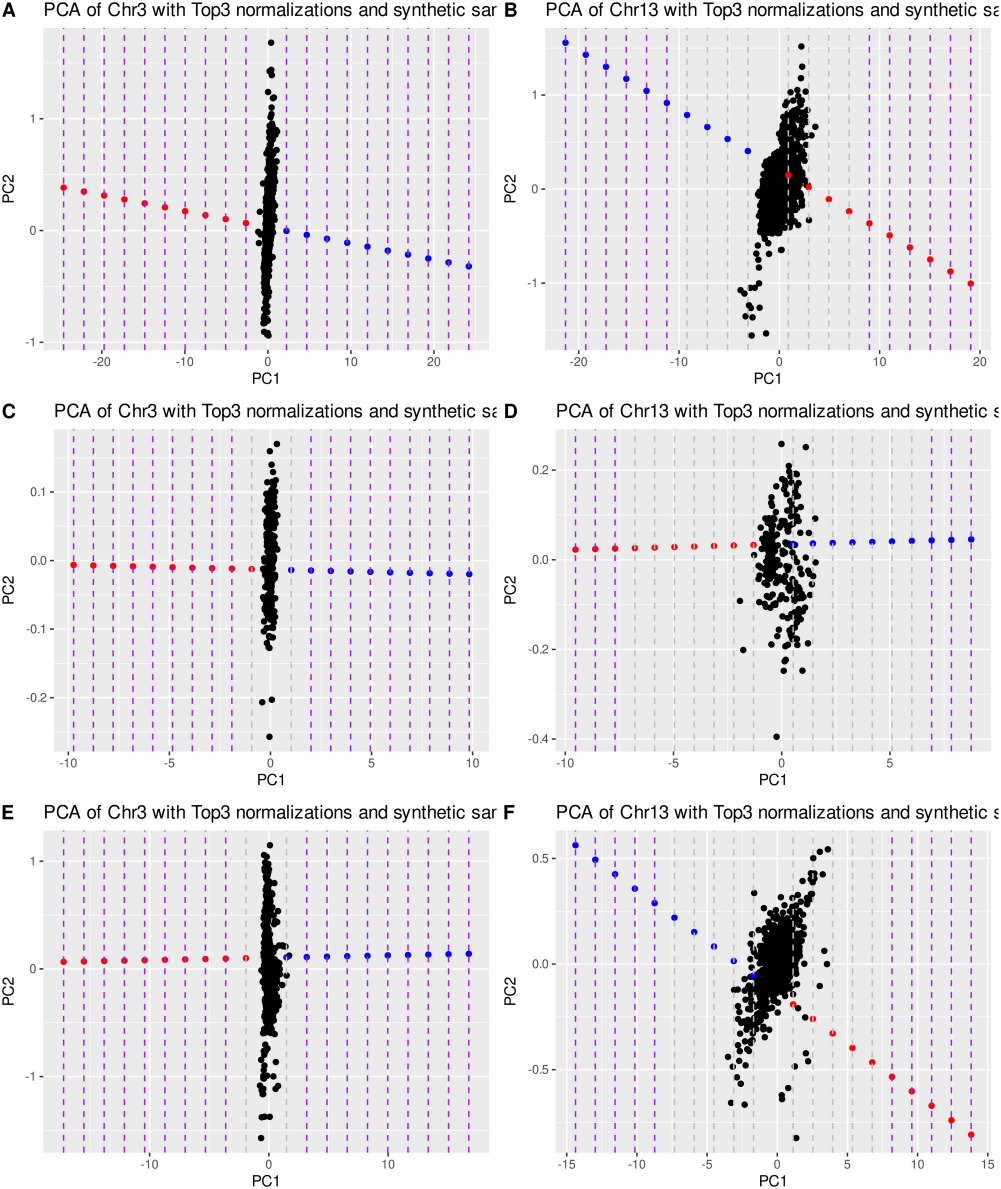
Chromosomal PCA plot showing simulated mosaic aneuploidy across different chromosomes. (A) Chr3 PCA of CES samples. (B) Chr13 PCA of CES samples. (C) Chr3 PCA of WES-SingleIndex samples. (D) Chr13 PCA of WES-SingleIndex samples. (E) Chr3 PCA of WES-DualIndex samples. (F) Chr13 PCA of WES-DualIndex samples. Synthetic samples with 10% coverage increase and 10% coverage reductions are shown in blue and red, respectively.

PCA plots of the three best normalizations for Chr3 and Chr13 and capture type are shown as examples in Fig. 3. With the threshold previously defined for each autosome (green mark Fig. 2), we delimited the significant region by the grey dotted lines, which indicate the percentage of syntenic aneuploidies in which both trisomy and monosomy present significant differences with respect to the control cohort. For Chr3, the best three normalizations correspond to Chr12, All, and Chr15 for CES (10% threshold, Fig. 3A), Chr12, Chr7, and All for WES-Sigle (20% threshold, Fig. 3C); and Chr12, Chr7, and Chr6 for WES-Dual (20% threshold, Fig. 3E). Meanwhile, for Chr13, the best three normalizations correspond to Chr4, Chr5, and Chr2 for CES (50% threshold, Fig. 3B), Chr5, Chr2, and Chr4 for WES-Sigle (80% threshold, Fig. 3D), and Chr4, Chr5, and Chr2 for WES-Dual (60% threshold, Fig. 3F).

**Figure 4:**
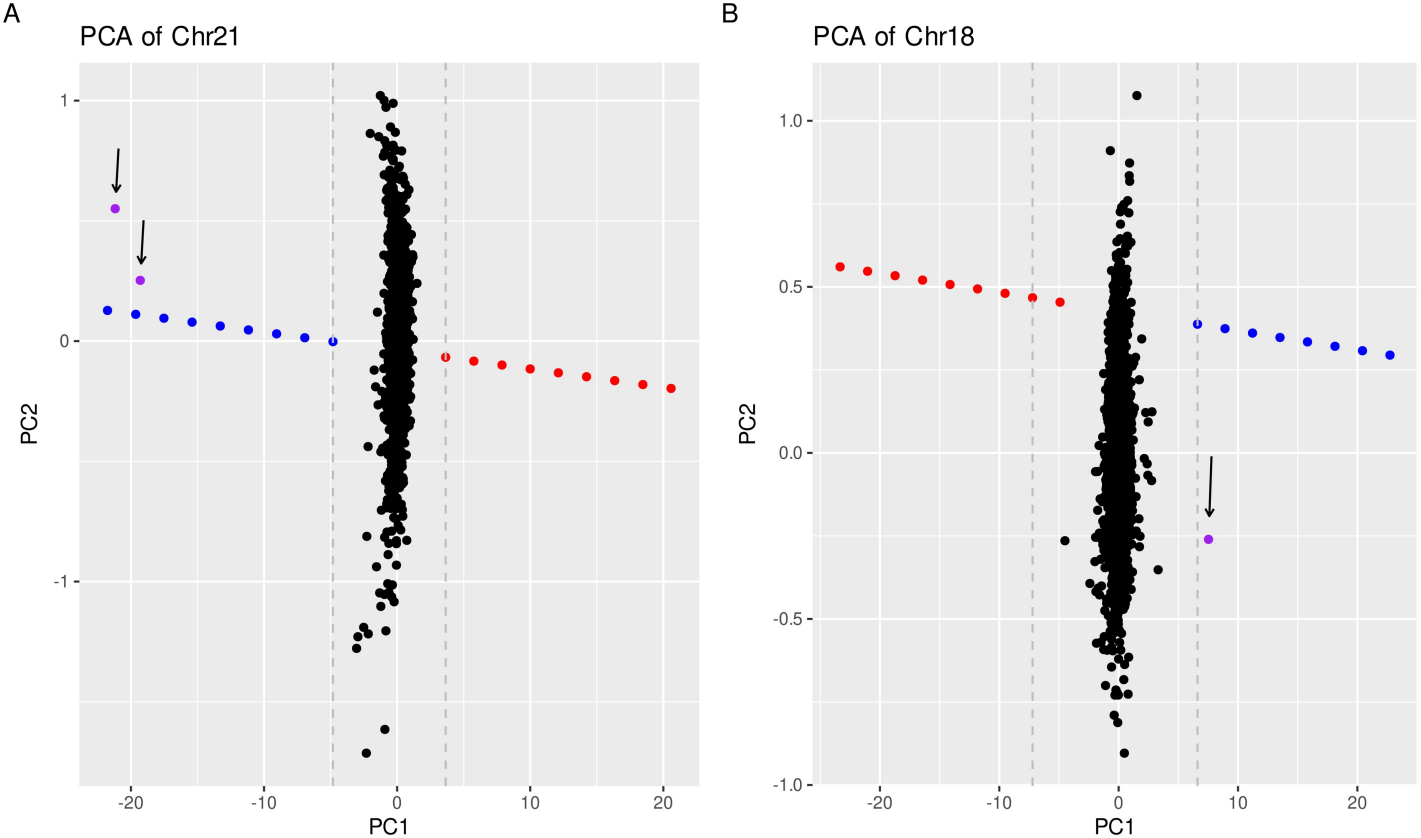
PCA detection of chromosome 21 and 18 Trisomies. (A) Chr21 PCA of the CES samples with 20% aneuploidy threshold (gray dotted line), with two validated outliers (arrows). (B) Chr18 PCA of the CES samples with 30% aneuploidy threshold (gray dotted line), with one validated outlier as a mosaic trisomy (arrow).

Once specific thresholds have been determined for each autosome using the control dataset, samples from the HSJD cohort were analysed. As shown in Fig. 4A, two clear outliers were detected on Chr21, corresponding to two Down syndrome patients, and in Fig. 4B a borderline outlier on Chr18 was identified. Chr18 outlier was less prominent than expected for a full trisomy. Thus, an orthogonal technique was used and a mosaic trisomy was confirmed. Autosomal outliers were validated by karyotyping or aCGH (Table 1).

**Table 1:** Autosome outliers validation by technique.

	Capture type	Kariotype	aCGH
Trisomy 21	CES	2	–
Mosaic Trisomy 18	CES	–	1

Additionally, we identified two outliers characterized by relatively low mean autosomal coverage (∼57×) and the presence of duplications on chromosomes 7 and 19. These events, which exceed 1 Mb in size, were subsequently validated using CNV detection tools (Figs. S6 and S7, available as supplementary data at *Bioinformatics* online). These findings highlight the method’s sensitivity in detecting not only complete aneuploidies but also more complex genomic alterations such as aneuploidies in mosaicism and large-scale copy number variants.

### 3.2 Sex chromosomes aneuploidies study

In order to visualize the samples with potential aneuploidies on sex chromosomes, normalized values for chromosomes X and Y were plotted for each of the evaluated normalizations. To determine the best normalizations and identify clear outliers that could indicate the presence of an aneuploidy on either of these sex chromosomes and infer the karyotype of the sample, beta-dispersion was calculated for the two main clusters corresponding to the XX and XY samples (Table 2). Beta-dispersion values obtained indicate that the normalizations with the lowest beta-dispersion values were reached with Chr2, Chr12, and Chr15 for the CES samples, with Chr3, Chr12, and Chr14 for the WES-Single samples, and with Chr3, Chr12, and Chr15 for the WES-Dual samples. The scatter plots of the three best normalizations for CES, WES-Single, and WES-Dual samples are presented in Fig. S8, available as supplementary data at *Bioinformatics* online.

**Table 2:** Beta-dispersion values of the XX and XY sample cluster for each chromosome normalization.

	CES			WES-Single			WES-Dual		
	XX_dispersion	XY_dispersion	mean_dispersion	XX_dispersion	XY_dispersion	mean_dispersion	XX_dispersion	XY_dispersion	mean_dispersion
Chr1	0.028	0.051	0.039	0.019	0.047	0.033	0.025	0.041	0.033
Chr2	0.016	0.051	0.033	0.047	0.057	0.052	0.037	0.037	0.037
Chr3	0.023	0.056	0.039	0.012	0.041	0.026	0.012	0.032	0.022
Chr4	0.029	0.063	0.046	0.056	0.062	0.059	0.055	0.047	0.051
Chr5	0.027	0.067	0.047	0.05	0.057	0.053	0.041	0.039	0.04
Chr6	0.023	0.062	0.043	0.034	0.051	0.043	0.024	0.033	0.029
Chr7	0.021	0.061	0.041	0.023	0.049	0.036	0.024	0.038	0.031
Chr8	0.023	0.061	0.042	0.033	0.053	0.043	0.03	0.04	0.035
Chr9	0.04	0.062	0.051	0.021	0.046	0.034	0.04	0.044	0.042
Chr10	0.22	0.061	0.041	0.044	0.057	0.05	0.031	0.037	0.034
Chr11	0.041	0.058	0.049	0.042	0.048	0.045	0.074	0.051	0.063
Chr12	0.022	0.056	0.039	0.018	0.044	0.031	0.016	0.033	0.024
Chr13	0.043	0.078	0.06	0.092	0.081	0.086	0.084	0.061	0.072
Chr14	0.028	0.053	0.041	0.018	0.043	0.031	0.024	0.039	0.031
Chr15	0.017	0.055	0.036	0.037	0.051	0.044	0.024	0.033	0.028
Chr16	0.075	0.087	0.081	0.097	0.071	0.084	0.129	0.079	0.104
Chr17	0.061	0.078	0.069	0.071	0.061	0.066	0.105	0.064	0.084
Chr18	0.027	0.065	0.046	0.066	0.064	0.065	0.064	0.048	0.056
Chr19	0.147	0.159	0.153	0.137	0.086	0.111	0.17	0.092	0.131
Chr20	0.063	0.08	0.071	0.07	0.057	0.063	0.109	0.063	0.086
Chr21	0.073	0.092	0.082	0.044	0.05	0.047	0.079	0.055	0.067
Chr22	0.084	0.095	0.09	0.097	0.067	0.082	0.141	0.075	0.108
Autosomes	0.025	0.057	0.041	0.021	0.046	0.033	0.028	0.039	0.033

**Figure 5:**
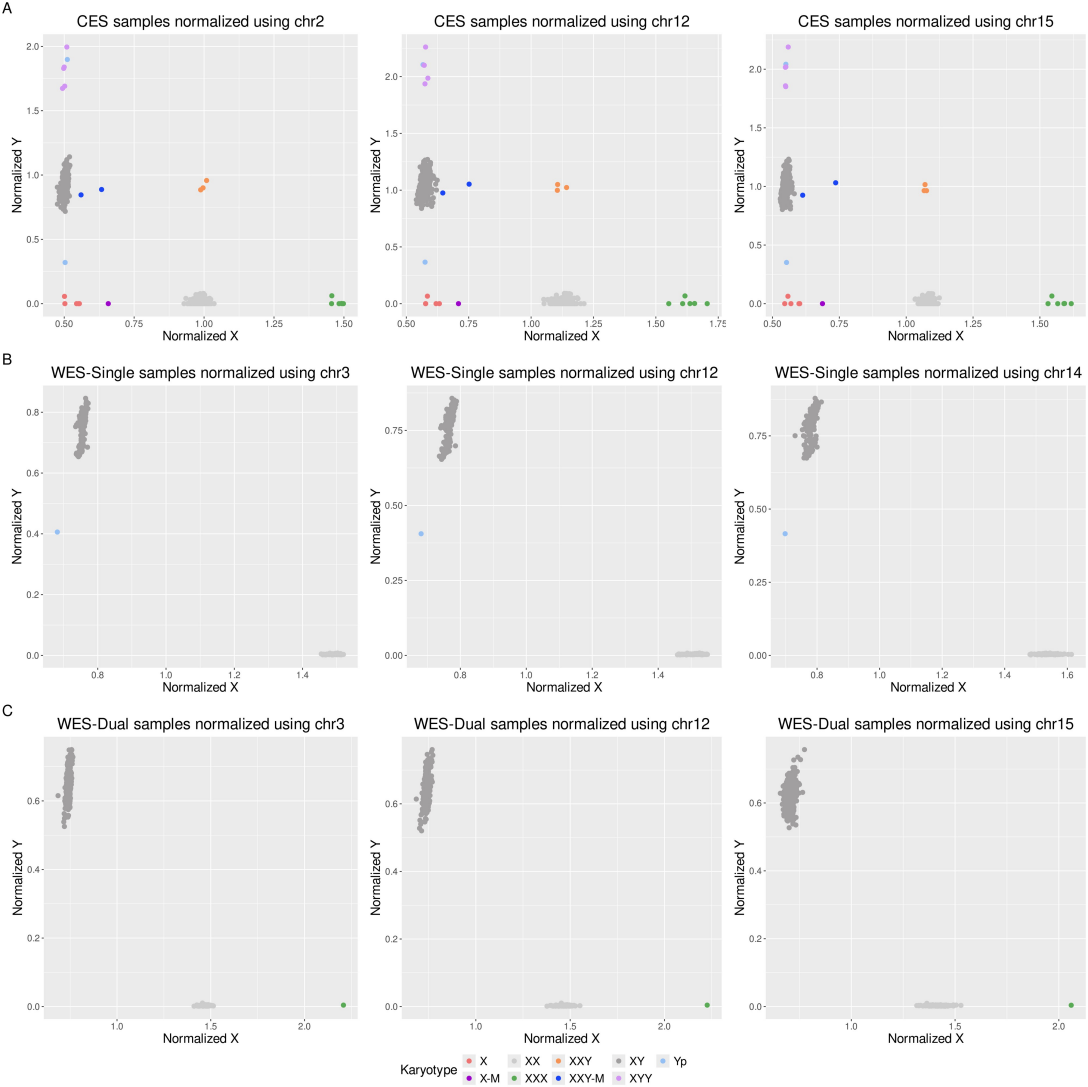
Scatterplots of sex chromosomes (X/Y) with best three normalizations. (A) CES samples with Chr2, Chr12, and Chr15 normalizations. (B) WES-SingleIndex samples with Chr3, Chr12, and Chr14 normalizations. (C) WES-DualIndex samples with Chr3, Chr12, and Chr15 normalizations.

Based on the three optimal normalizations for each type of data, the HSJD cohort samples were analysed with respect to the control cohort. Samples with XX and XY karyotypes form two main clusters, as shown in Fig. 5 (grey dots). The XX sample cluster is centered between 1 and 1.5 for the normalized X values, with values closer to 1 for the CES samples and values closer to 1.5 for the WES samples. Meanwhile the normalized Y values are centered around 0 normalized X values across all data types. On the other hand, the XY sample cluster were centered between 0.6 and 1 for the normalized Y values, with values closer to 0.6 for the WES-Dual samples, values closer to 0.8 for the WES-Single samples, and values closer to 1 for the CES samples. Normalized X values were closer to 0.5 for the CES and WES-Dual samples and closer to 0.8 for the WES-Single samples.

Multiple clear and significant outliers were detected (ADONIS2 tests *P*-value <.01). From the 6611 analysed samples seven Triple X syndrome (XXX), six Turner syndrome (X0), five Klinefelter syndrome (XXY), and five XYY samples were identified. From these, three samples were detected in mosaicism, two Klinefelter syndrome and one Turner syndrome. The XXY sample closest to the control XY group had a mosaicism rate of 13% (validated by aCGH, Karyotype, and FISH). Although the mosaic aneuploidies did not show as significant differences with an ADONIS2 test both XXY samples were significant at a *P*-value <.05. Moreover, mosaic samples, although not significant, deviated from the control groups in a stable manner with all three best normalizations. Six samples with average Y coverage showed values greater than 1.5 in CES. Five of these samples correspond to an XYY karyotype and one had a partial Y chromosome duplication determined by aCGH (shown in light blue color, Fig. 5A). Finally, one sample had a faint signal for ChrY, higher than Turner syndrome samples and lower than normal XY samples. After aCGH validation, a partial deletion of Y chromosome was confirmed.

All outliers except one were confirmed to come from individuals with sex chromosomes aneuploidies detected by orthogonal techniques, as shown in Table 3. One Turner syndrome sample and low Klinefelter mosaic sample were validated by more than one orthogonal technique.

**Table 3:** Sex chromosome outliers validation by technique.

Names	Capture type	Samples	Kariotype	aCGH	FISH	QF-PCR	Non verified
XXX	CES	6	2	1	–	3	–
	WES-Dual	1	–	1	–	–	–
X	CES	5	4	1	1	–	–
XXY	CES	3	3	–	–	–	–
XYY	CES	5	4	–	–	1	–
XXY-M^a^	CES	2	1	1	1	1	–
X-M^a^	CES	1	–	–	–	1	–
Yp	CES	2	–	2	–	–	–
	WES-Single	1	–	–	–	–	1

a M = Mosaicism.

Overall, these findings demonstrate the robustness of our method in detecting a wide range of sex chromosome aneuploidies, including mosaic cases.

## 4 Discussion and conclusions

In this study, we prove the use of NGS data to determine chromosomal aneuploidies in a precise manner. We have developed a method that provides a complete and accurate study of aneuploidies for autosomal as well as for sex chromosomes.

Mean coverage measure represents a major advantage over commonly used tools/metrics for aneuploidy/sex identification. Commonly used tools are not specifically designed for this purpose, making their results less specific. An example of this is Peddy (Pedersen and Quinlan 2017), a tool created for the detection and correction of errors related to sex, relatedness, or ancestry. Peddy allows the identification of X-linked sex aneuploidies using the ratio between heterozygous and homozygous genotypes on the X chromosome used to predict sex. Nevertheless, most of the aneuploidies detected in this study would have been missed using this kind of approach. Other tools used in noninvasive prenatal testing (NIPT) as WisecondorX (Raman *et al.* 2019) or NIPTeR (Johansson *et al.* 2018) are less intuitive and in many cases computationally demanding compared with the methodology presented in this article.

Using PCA with coverage values from samples normalized by the top three best methods allows for the detection of both complete and mosaic aneuploidies in autosomes, as seen in this study for trisomy 21 and mosaic trisomy 18. With the usage of synthetic samples, we determined the minimum percentage of mosaic aneuploidy detection per chromosome. Although the results demonstrate an accurate detection of aneuploidies with mosaicism percentages exceeding 20% in almost all autosomes, it was also observed that samples with large duplications or deletions could be detected as clear outliers, potentially leading to confusion with true aneuploidies. Therefore, it is necessary to rule out the presence of structural variants (SVs) that might interfere with individual analysis, by plotting the chromosome coverage compared with the control samples (Fig. S7, available as supplementary data at *Bioinformatics* online) or using general CNV callers.

As a caveat, if a sample has complete or mosaic aneuploidy in an autosome used for normalizing another, it may affect the latter’s distribution to varying degrees. Thus, it is essential to review all autosomes for abnormal distributions, as the sample will show a clearer deviation in the altered chromosome and a milder one in autosomes normalized against it. This effect can be observed in Fig S9, available as supplementary data at *Bioinformatics* online (Down syndrome samples exhibit a deviation at Chr17 when is normalized using Chr21). For this reason, chromosomes 13, 18, and 21 were excluded from the top three normalizing chromosomes, as they are more likely to exhibit aneuploidies.

The usage of scatter plots that integrate the normalized values of both sex chromosomes allows a clear identification of samples with complete or mosaic aneuploidies. We have been able to clearly identify samples with Turner syndrome, Klinefelter syndrome, Trisomy X syndrome, and XYY syndrome. Due to the biased coverage seen in ChrY captured regions (Fig. S2, available as supplementary data at *Bioinformatics* online) alterations that do not involve this region could remain undetected. In contrast, as seen with the sample showing a partial Y duplication, if the duplication overlaps the covered region, it may be misidentified as an XYY sample.

Interestingly, some mosaic aneuploidies were also detected although with *P*-values not statistically significant. This result indicates that the present model allows the detection of subtle aneuploidies, although these would still require a more manual inspection. Therefore, it is always important to carefully review the sample quality, ensure that the alteration is consistent across multiple normalizations, and verify that the sample does not present any other alterations that could influence its distribution in the plots.

Although this strategy has proven to be highly precise, it is important to validate any potential aneuploidies specially in ChrY. Specific orthologous techniques, such as karyotyping, array CGH, QF-PCR, or FISH, are required to identify possible false positive determinations.

In comparison with other widely used coverage-based callers such as ExomeDepth (Plagnol *et al.* 2012), XHMM (Fromer *et al.* 2012), or GATK gCNV (Van der Auwera and O’Connor 2020), which employ panel-of-normals or window-based approaches to identify subchromosomal copy number changes, our method offers a distinct operational advantage for large-scale screenings. While these established tools are highly effective for identifying focal CNVs by analysing read-depth variations across specific genomic bins or exons, they are not primarily optimized for the rapid detection of whole-chromosome or mosaic aneuploidies. Furthermore, these algorithms often require substantial computational resources, complex normalization pipelines, and extensive parameter tuning to account for systematic noise across thousands of small intervals.

In conclusion, based on the comprehensive set of findings obtained in this study, the use of NGS data allows the detection of complete and mosaic aneuploidies. This approach allows the detection of aneuploidies in cases where there is no clear clinical suspicion of the alteration, and for which the specific test would not initially be requested and can be particularly useful for conditions with low diagnostic rates at an early age, such as Klinefelter syndrome.

## Supplementary Material

btag104_Supplementary_Data

## Data Availability

A detailed guide is available in the GitHub repository (https://github.com/B-R-I-D-G-E/AneuploidiesStudies), which provides instructions for using multiple scripts that enable: (1) the generation of an RData file containing the coverage values required for the analyses, (2) the execution of a control cohort study, and (3) the analysis of pool samples based on the results of the control cohort study. The version used in this study is archived 10.5281/zenodo.18363291.
