# Supplementary material for: Identification of autosomal and sex chromosome aneuploidies using next generation sequencing
Source: Bioinformatics. 2026 Mar 16;42(3):btag104. doi: 10.1093/bioinformatics/btag104 (PMC13032822; doi:10.1093/bioinformatics/btag104)
Supplement: btag104_Supplementary_Data [file btag104_supplementary_data.zip › Paper_aneuploidies_supp.pdf]

SUPPLEMENTARY FIGURES:

Supp Figure 1: Density plots of mean coverage values for autosomes of control and cohort datasets A) control CES samples B) control WES-Single Index samples C) control WES-Dual Index samples D) cohort CES samples. E) cohort WES-Single Index samples. F) cohort WES-Dual Index samples.

Supp Figure 2: Capture regions used in this study from CES and WES A) All chromosomes circos capture plot B) Density plot of ChrY capture regions.

Supp Figure 3: Best three normalizations excluding those performed with Chr13, Chr18 and Chr21 for each autosome with the CES samples and with synthetic samples simulating mosaic aneuploidies at different percentages 10-100% in each autosome.

Supp Figure 4: Best three normalizations excluding those performed with Chr13, Chr18 and Chr21 for each autosome with the WES-Single Index samples and with synthetic samples simulating mosaic aneuploidies at different percentages 10-100% in each autosome.

Supp Figure 5: Best three normalizations excluding those performed with Chr13, Chr18 and Chr21 for each autosome with the WES-Dual Index samples and with synthetic samples simulating mosaic aneuploidies at different percentages 10-100% in each autosome.

Supp Figure 6: PCA of Chr7 and Chr19 from the WES-Dual Index samples with the synthetical samples (detected as clear outliers), and with two outliers corresponding to samples with coverage close to the minimum quality threshold and with the presence of CNVs on chromosome 7 and 19.

Supp Figure 7: Density plot of read number by position on Chr7 (large CNV duplication). Altered sample (purple) compared to normal distribution in the control cohort (grey).

Supp Figure 8: Scatter-plots with the coverage values of the X and Y chromosomes normalised for the three best normalizations for the CES, WES Single and WES Dual Index samples respectivelly.

Supp Figure 9: Example of samples displacement in the PCA of Chr17 without alteration, due to the presence of aneuploidies in Chr21, used as one of the three selected normalizations to construct the PCA.

Supp Figure 1.

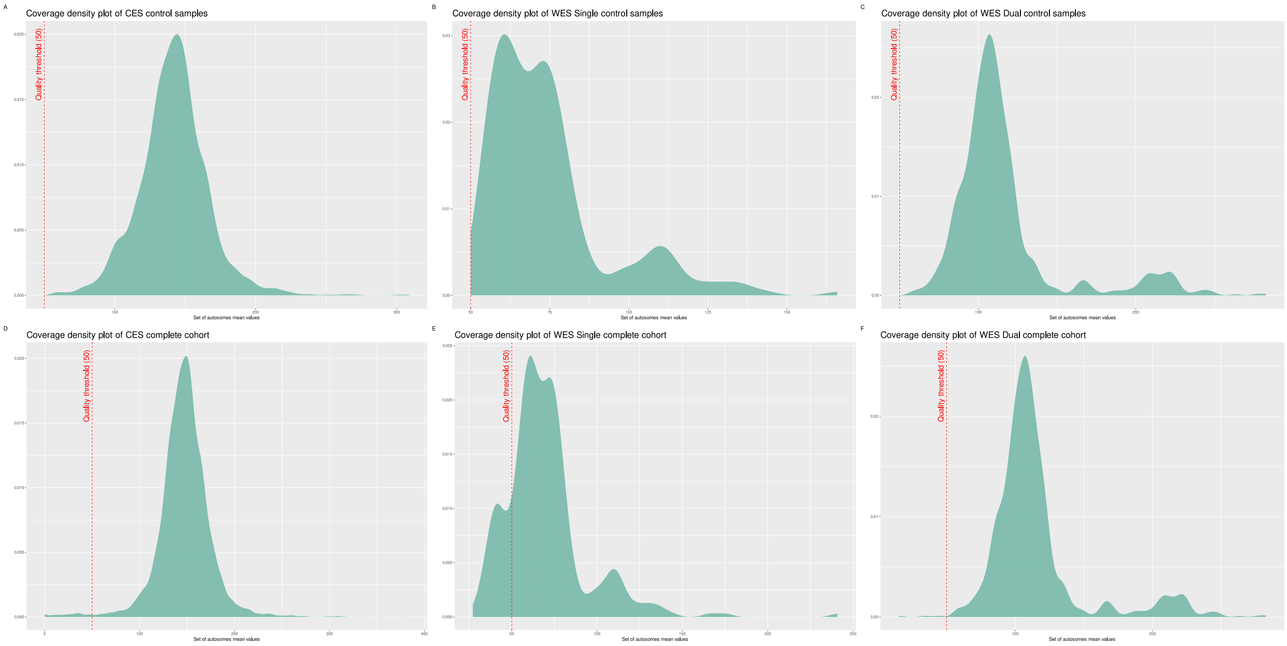

Supp Figure 2.

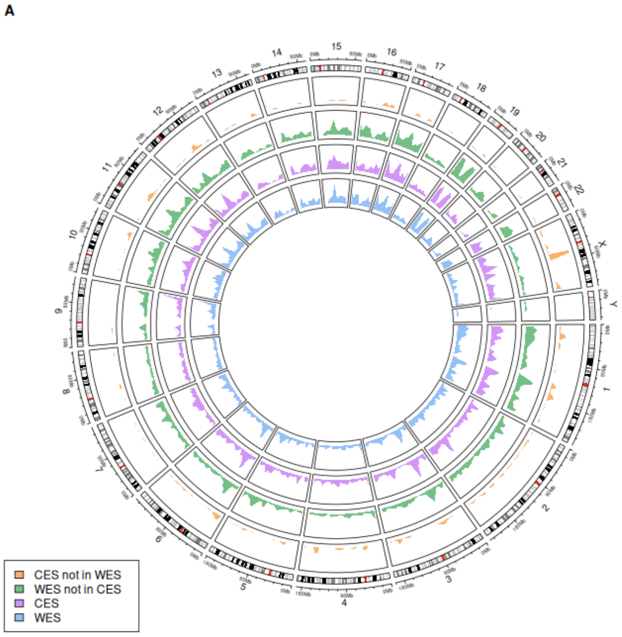

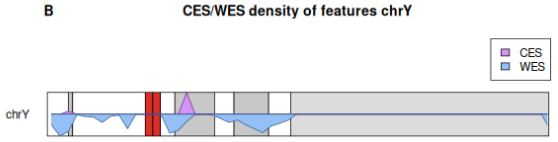

Supp Figure 3.

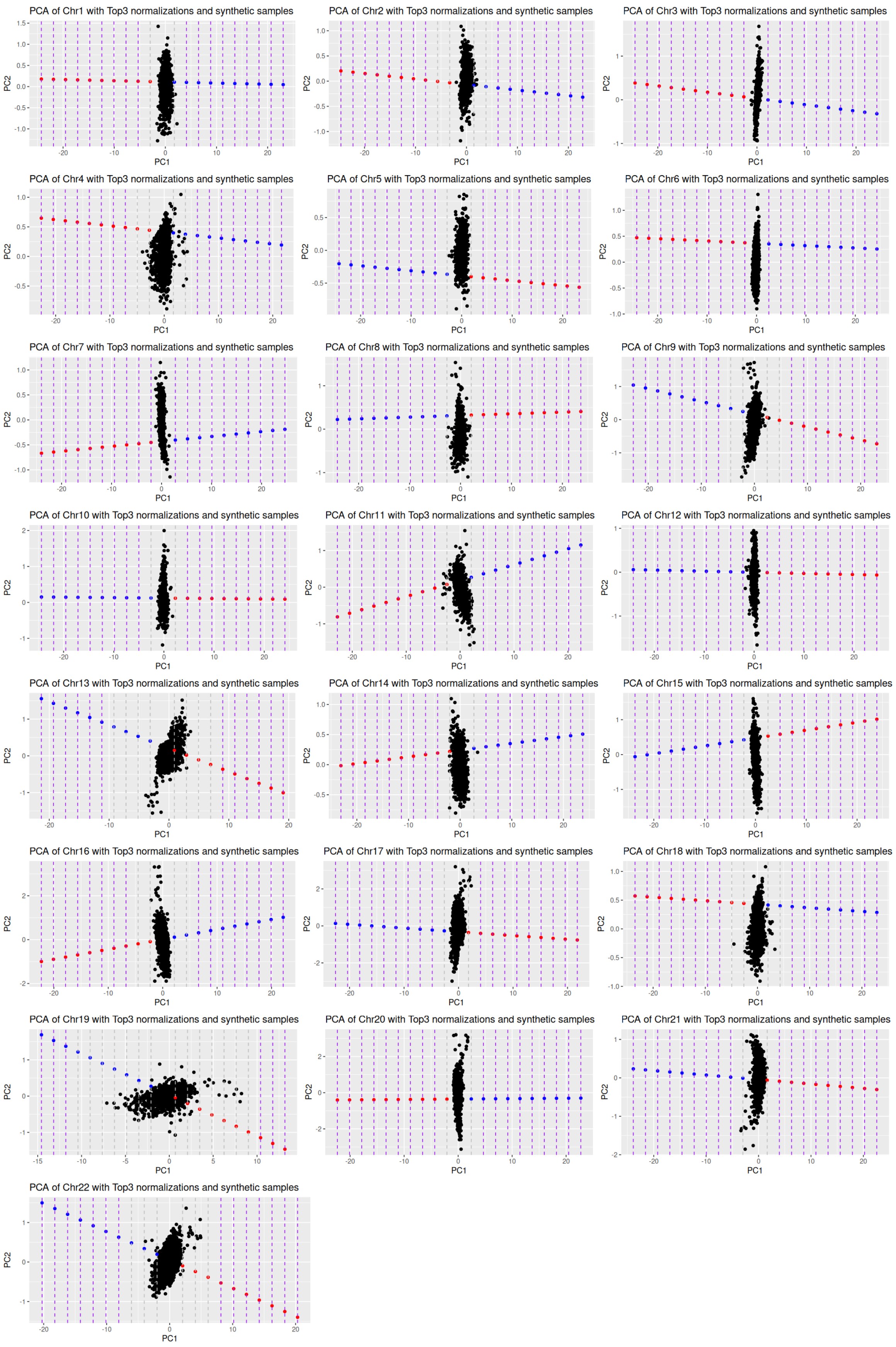

Supp Figure 4.

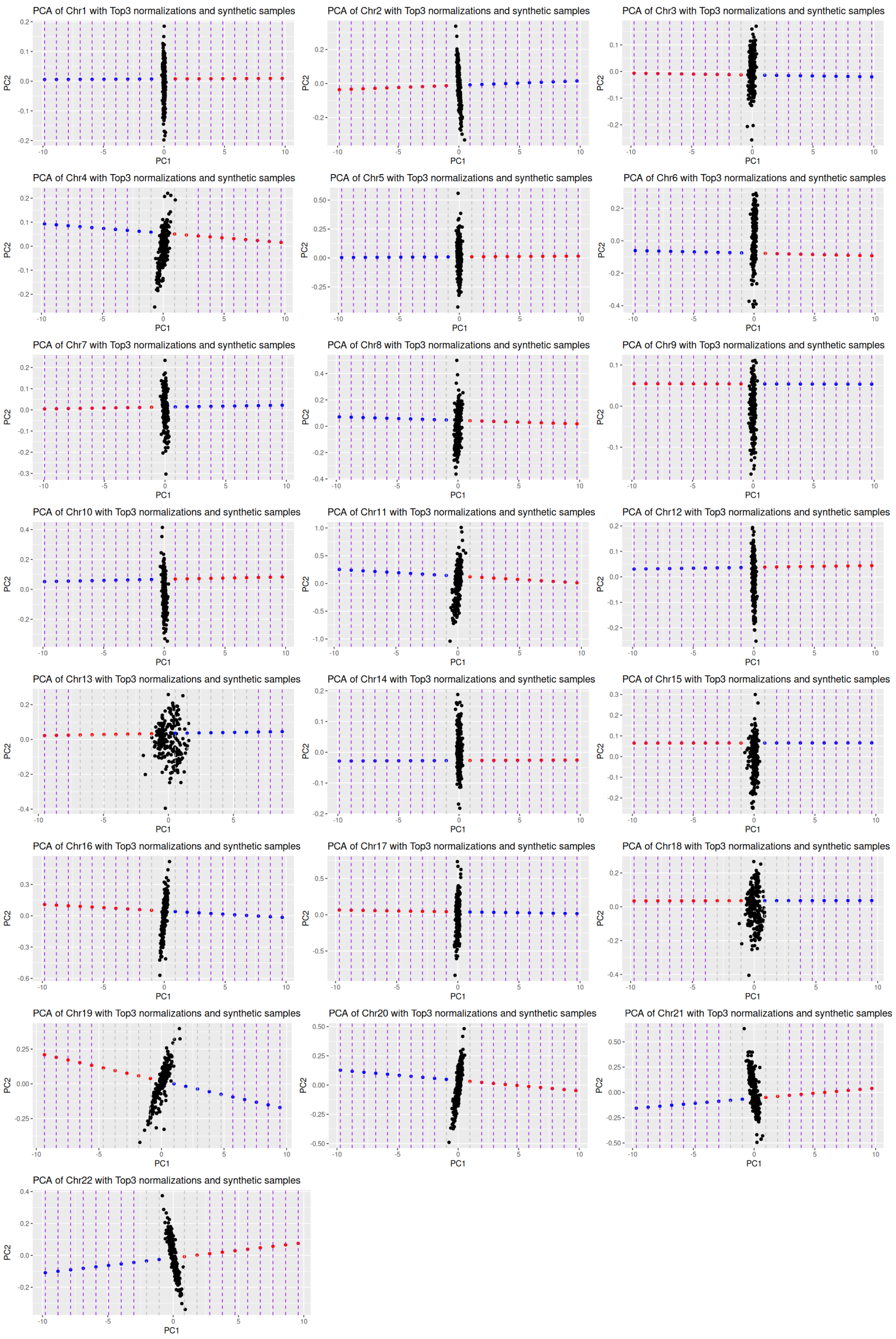

Supp Figure 5.

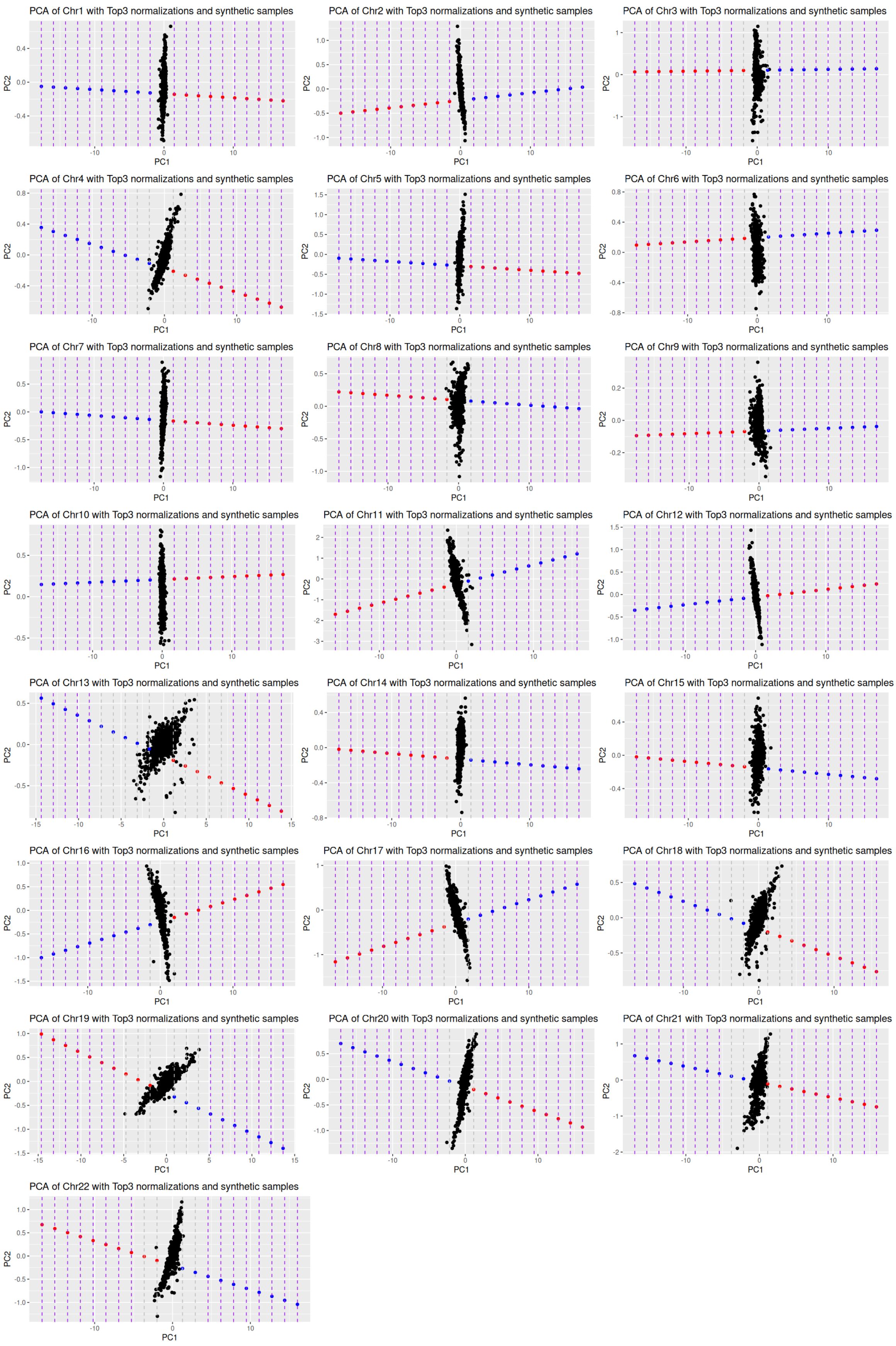

Supp Figure 6.

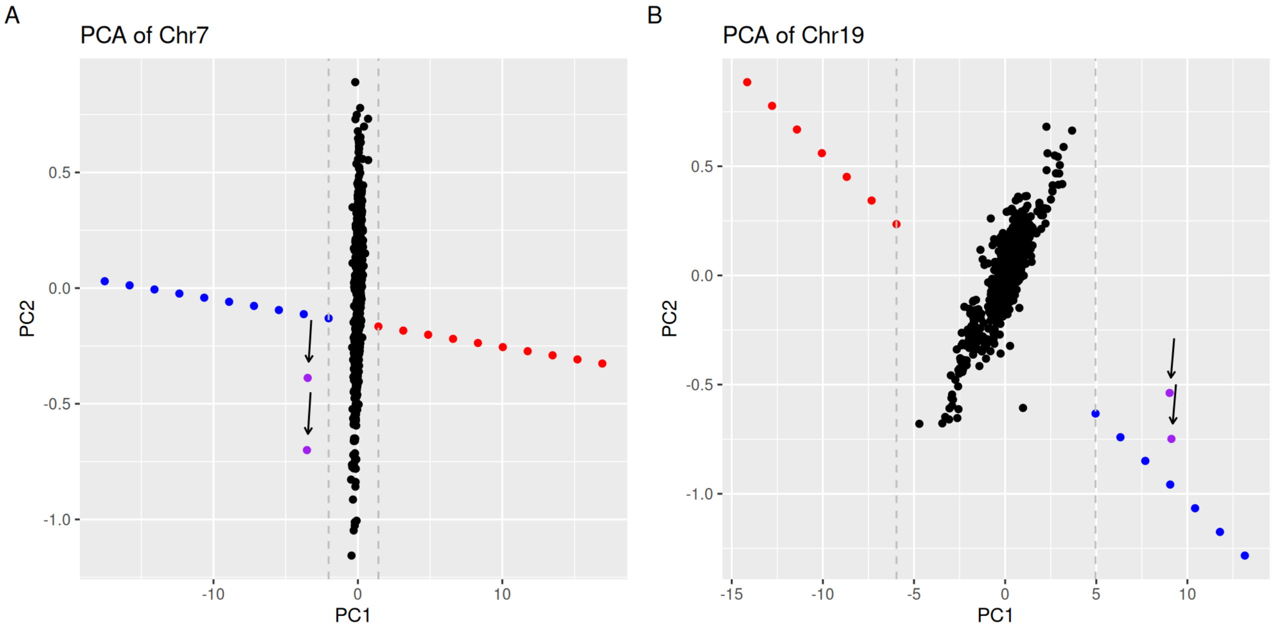

Supp Figure 7.

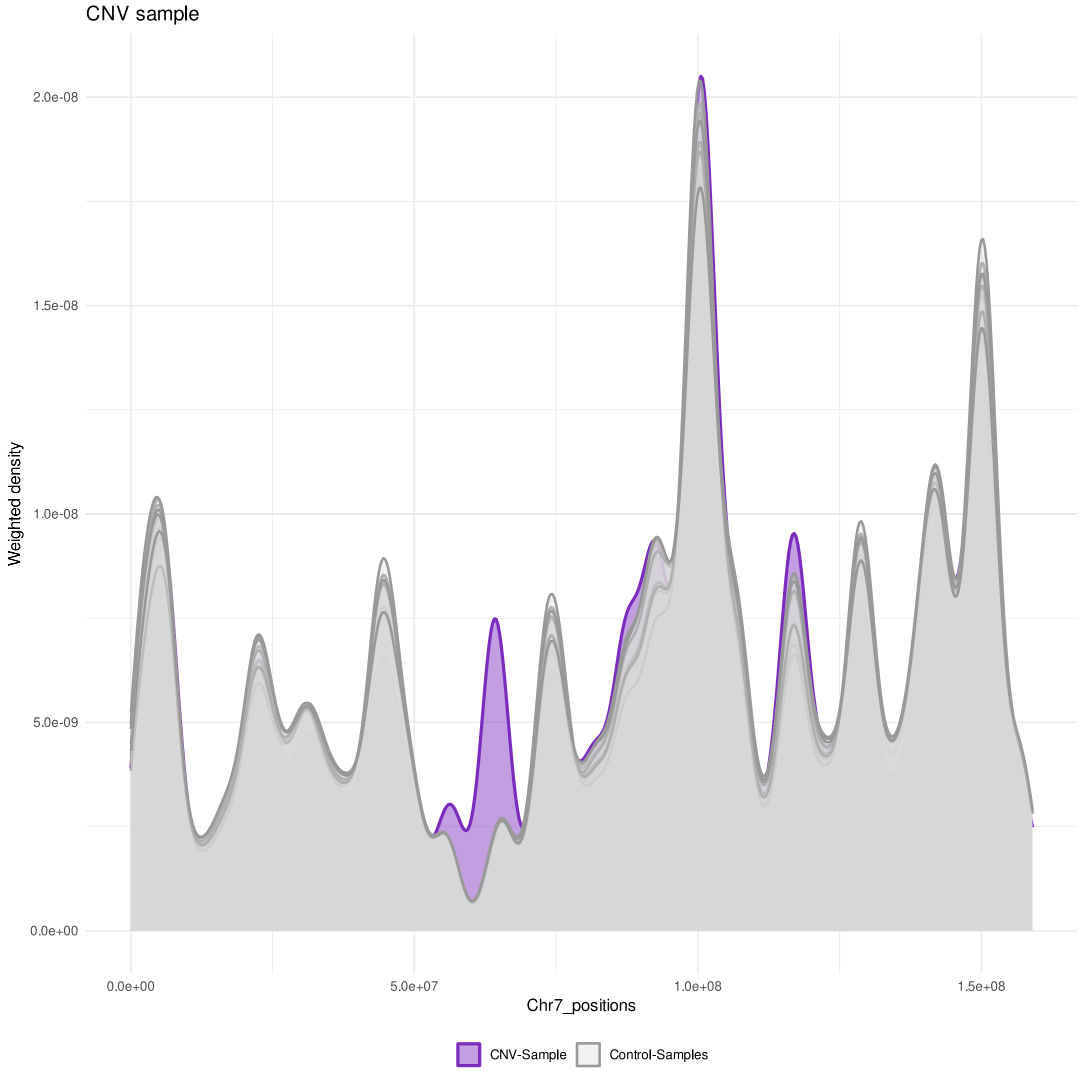

Supp Figure 8.

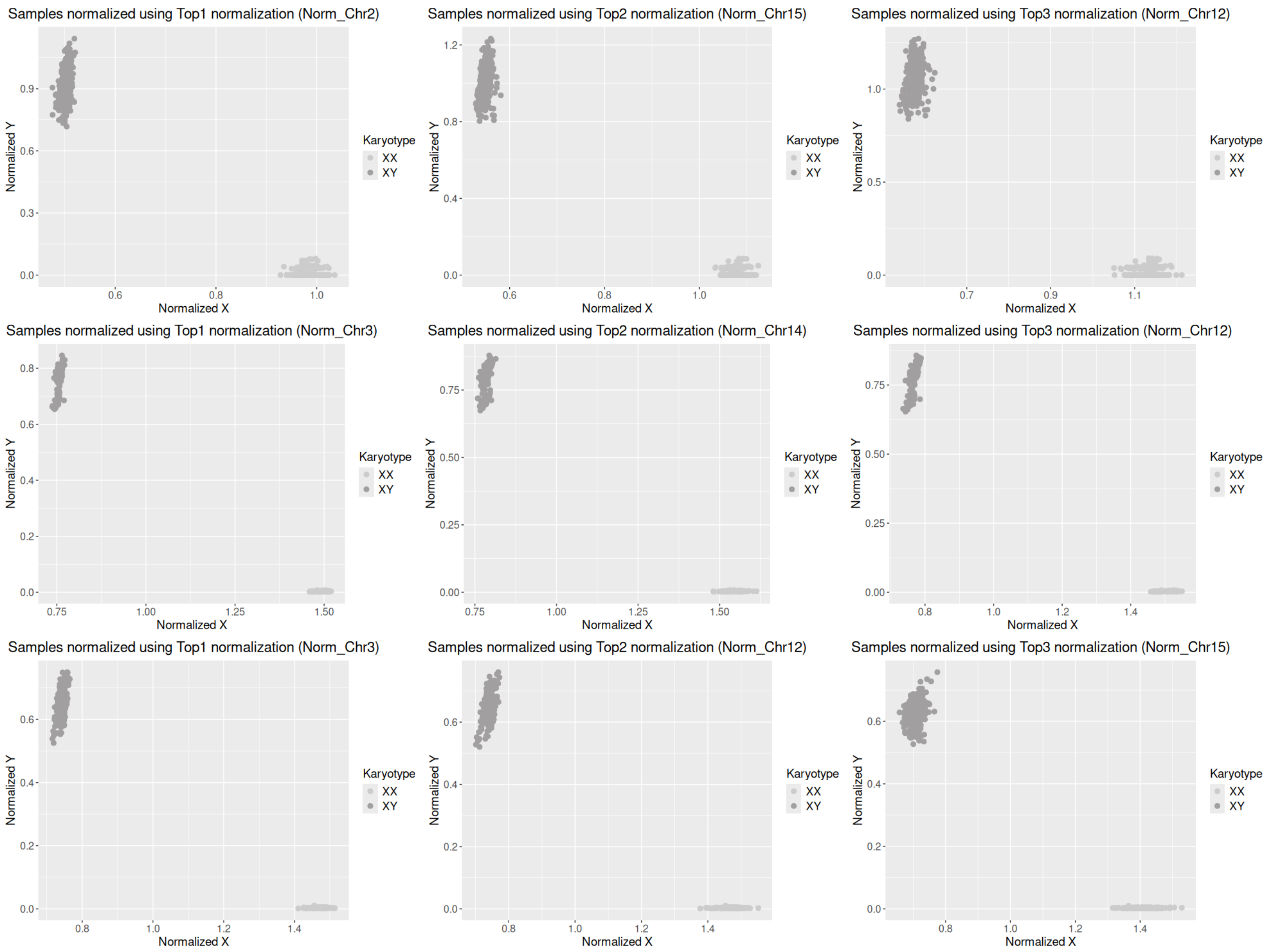

Supp Figure 9.

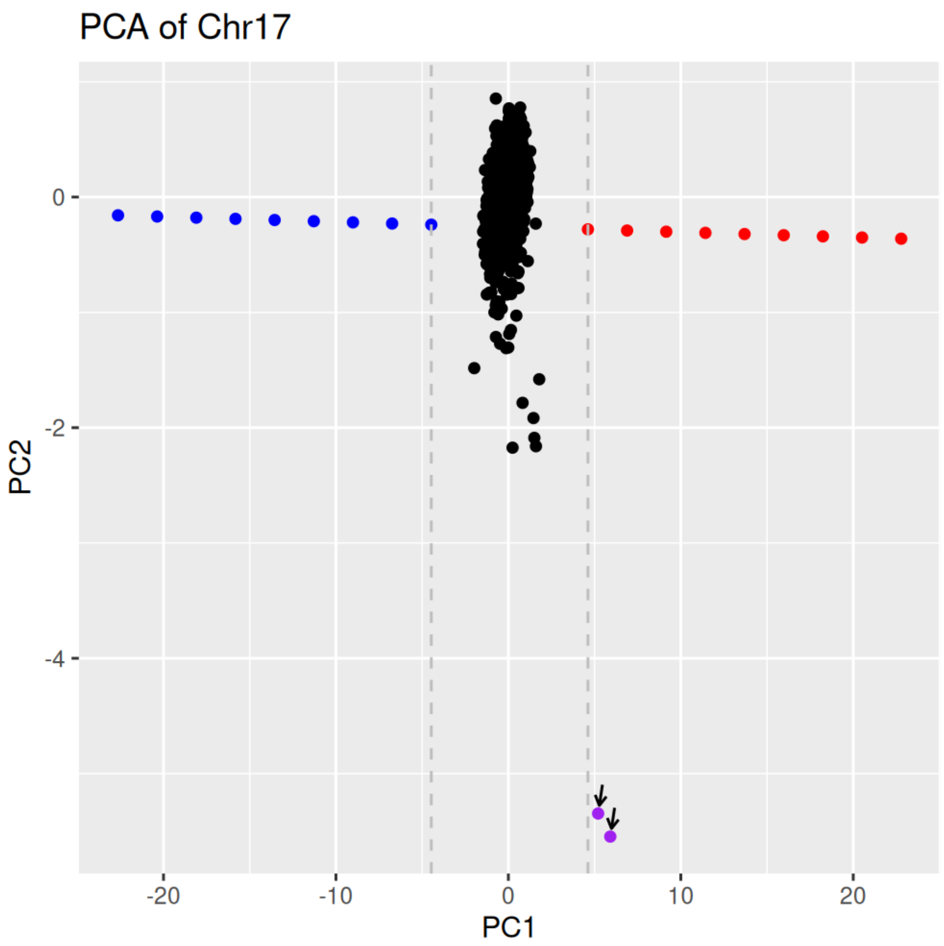

SUPPLEMENTARY TABLES

Supp Table 1: Summarized coverage distribution in Controls and HSJD analyzed cohort.

Supp Table 2: Coverage confident range for HSJD cohort.

Supp Table 3: Kluskal Test (p-value) comparing captures and indexes for each autosome.

Supp Table 4: Standard deviation values for each autosome and each normalization for the CES, WES-Single Index and WES-Dual Index samples.

Supp Table 1.

	Controls			HSJD cohort		
	**CES**	**WES-Single**	**WES-Dual**	**CES**	**WES-Single**	**WES-Dual**
Mean	146.77	69.49	115.33	141.79	74.49	116.33
Median	147.20	64.86	107.70	141.66	70.73	107.89
1st quartile	133.36	56.18	97.62	127.98	60.43	98.15
3rd quartile	161.01	77.11	119.14	154.80	78.54	119.45

Supp Table 2.

Coverage	Z-score
10	-4.737
20	-4.391
30	-4.044
40	-3.698
50	-3.352
60	-3.005
70	-2.659
80	-2.313
90	-1.966
100	-1.620

Supp Table 3.

Chromosome	CES Vs WES (p-value)	WES-Single Vs WES-Dual (p-value)
Chr1	0.00E+00	2.08E-01
Chr2	0.00E+00	2.18E-71
Chr3	5.88E-90	5.30E-93
Chr4	0.00E+00	1.18E-75
Chr5	4.10E-08	4.03E-76
Chr6	4.74E-17	2.05E-66
Chr7	3.41E-312	1.63E-62
Chr8	0.00E+00	5.33E-23
Chr9	0.00E+00	4.65E-14
Chr10	3.39E-175	6.29E-69
Chr11	2.12E-33	1.46E-58
Chr12	2.72E-215	1.14E-62
Chr13	1.26E-61	1.03E-54
Chr14	0.00E+00	5.62E-19
Chr15	1.28E-53	1.83E-87
Chr16	9.53E-87	4.79E-76
Chr17	8.43E-207	4.19E-82
Chr18	1.28E-212	2.03E-73
Chr19	0.00E+00	3.86E-83
Chr20	0.00E+00	8.35E-70
Chr21	0.00E+00	3.23E-18
Chr22	3.94E-275	2.30E-66

Supp Table 4.

CES																						
	Chr1	Chr2	Chr3	Chr4	Chr5	Chr6	Chr7	Chr8	Chr9	Chr10	Chr11	Chr12	Chr13	Chr14	Chr15	Chr16	Chr17	Chr18	Chr19	Chr20	Chr21	Chr22
**Norm_Chr1**	NA	0,040	0,012	0,055	0,044	0,036	0,033	0,030	0,014	0,031	0,017	0,014	0,069	0,013	0,022	0,046	0,036	0,041	0,073	0,032	0,034	0,055
**Norm_Chr2**	0,034	NA	0,023	0,018	0,012	0,009	0,009	0,016	0,041	0,013	0,047	0,021	0,031	0,035	0,015	0,072	0,061	0,013	0,091	0,057	0,056	0,079
**Norm_Chr3**	0,013	0,030	NA	0,047	0,034	0,027	0,023	0,021	0,022	0,021	0,028	0,005	0,061	0,017	0,012	0,058	0,046	0,033	0,084	0,042	0,043	0,067
**Norm_Chr4**	0,054	0,021	0,042	NA	0,015	0,019	0,023	0,032	0,061	0,027	0,069	0,040	0,020	0,056	0,034	0,094	0,083	0,015	0,112	0,077	0,075	0,102
**Norm_Chr5**	0,047	0,015	0,033	0,017	NA	0,009	0,012	0,020	0,053	0,015	0,061	0,031	0,026	0,047	0,024	0,087	0,075	0,013	0,107	0,070	0,068	0,095
**Norm_Chr6**	0,040	0,011	0,027	0,021	0,009	NA	0,008	0,015	0,047	0,010	0,054	0,025	0,032	0,039	0,018	0,081	0,069	0,013	0,102	0,064	0,063	0,090
**Norm_Chr7**	0,036	0,012	0,023	0,025	0,012	0,008	NA	0,010	0,043	0,007	0,050	0,021	0,037	0,035	0,014	0,077	0,064	0,017	0,099	0,060	0,059	0,085
**Norm_Chr8**	0,031	0,019	0,019	0,032	0,019	0,014	0,009	NA	0,037	0,010	0,043	0,019	0,041	0,028	0,013	0,068	0,056	0,024	0,090	0,053	0,051	0,077
**Norm_Chr9**	0,016	0,057	0,024	0,072	0,059	0,051	0,047	0,043	NA	0,044	0,012	0,026	0,087	0,021	0,037	0,039	0,030	0,055	0,071	0,024	0,027	0,050
**Norm_Chr10**	0,033	0,016	0,020	0,028	0,015	0,009	0,007	0,011	0,039	NA	0,047	0,018	0,039	0,032	0,012	0,073	0,061	0,017	0,095	0,057	0,056	0,081
**Norm_Chr11**	0,018	0,059	0,028	0,074	0,061	0,054	0,049	0,045	0,011	0,048	NA	0,030	0,088	0,021	0,040	0,033	0,023	0,059	0,064	0,019	0,023	0,042
**Norm_Chr12**	0,015	0,028	0,005	0,045	0,033	0,026	0,022	0,020	0,024	0,019	0,031	NA	0,059	0,019	0,011	0,060	0,049	0,031	0,087	0,044	0,045	0,069
**Norm_Chr13**	0,069	0,037	0,055	0,020	0,024	0,030	0,034	0,041	0,073	0,037	0,081	0,053	NA	0,068	0,046	0,104	0,093	0,029	0,119	0,087	0,083	0,112
**Norm_Chr14**	0,013	0,039	0,015	0,053	0,042	0,035	0,031	0,027	0,017	0,030	0,019	0,016	0,065	NA	0,023	0,046	0,035	0,041	0,071	0,031	0,033	0,055
**Norm_Chr15**	0,023	0,018	0,011	0,035	0,023	0,017	0,013	0,013	0,032	0,011	0,038	0,010	0,048	0,024	NA	0,065	0,053	0,023	0,088	0,049	0,049	0,073
**Norm_Chr16**	0,061	0,110	0,068	0,123	0,106	0,097	0,092	0,088	0,044	0,091	0,040	0,070	0,137	0,062	0,083	NA	0,019	0,102	0,042	0,017	0,016	0,018
**Norm_Chr17**	0,045	0,090	0,052	0,105	0,087	0,080	0,075	0,070	0,032	0,073	0,027	0,054	0,117	0,046	0,065	0,018	NA	0,086	0,051	0,011	0,012	0,026
**Norm_Chr18**	0,047	0,018	0,034	0,017	0,014	0,014	0,018	0,027	0,053	0,019	0,063	0,031	0,033	0,049	0,026	0,090	0,078	NA	0,111	0,072	0,071	0,099
**Norm_Chr19**	0,157	0,228	0,163	0,241	0,212	0,202	0,194	0,190	0,130	0,194	0,127	0,165	0,256	0,159	0,185	0,070	0,087	0,209	NA	0,088	0,078	0,055
**Norm_Chr20**	0,043	0,092	0,052	0,106	0,089	0,081	0,076	0,071	0,028	0,074	0,024	0,054	0,120	0,045	0,066	0,018	0,012	0,086	0,056	NA	0,011	0,030
**Norm_Chr21**	0,055	0,105	0,062	0,120	0,101	0,092	0,086	0,081	0,036	0,084	0,034	0,064	0,134	0,054	0,077	0,019	0,015	0,098	0,057	0,012	NA	0,032
**Norm_Chr22**	0,075	0,127	0,082	0,140	0,121	0,113	0,107	0,104	0,058	0,106	0,054	0,084	0,154	0,078	0,098	0,018	0,029	0,118	0,035	0,030	0,027	NA
**Norm_all**	0,012	0,034	0,006	0,050	0,037	0,030	0,025	0,021	0,020	0,023	0,025	0,009	0,063	0,012	0,016	0,054	0,042	0,036	0,080	0,038	0,039	0,063

WES-Single																						
	Chr1	Chr2	Chr3	Chr4	Chr5	Chr6	Chr7	Chr8	Chr9	Chr10	Chr11	Chr12	Chr13	Chr14	Chr15	Chr16	Chr17	Chr18	Chr19	Chr20	Chr21	Chr22
**Norm_Chr1**	NA	0,040	0,012	0,044	0,045	0,028	0,012	0,021	0,005	0,034	0,023	0,012	0,082	0,006	0,036	0,048	0,038	0,068	0,070	0,041	0,024	0,057
**Norm_Chr2**	0,033	NA	0,029	0,011	0,004	0,011	0,024	0,018	0,038	0,007	0,055	0,026	0,038	0,035	0,012	0,074	0,067	0,023	0,094	0,071	0,054	0,085
**Norm_Chr3**	0,010	0,030	NA	0,033	0,034	0,019	0,009	0,015	0,014	0,025	0,030	0,006	0,069	0,010	0,026	0,053	0,045	0,055	0,073	0,047	0,030	0,062
**Norm_Chr4**	0,043	0,013	0,036	NA	0,013	0,020	0,033	0,027	0,048	0,018	0,066	0,035	0,040	0,044	0,023	0,087	0,079	0,024	0,108	0,083	0,065	0,099
**Norm_Chr5**	0,035	0,004	0,031	0,011	NA	0,014	0,026	0,020	0,040	0,009	0,057	0,028	0,033	0,037	0,014	0,075	0,068	0,018	0,094	0,071	0,056	0,086
**Norm_Chr6**	0,024	0,012	0,018	0,018	0,016	NA	0,014	0,009	0,028	0,008	0,046	0,015	0,050	0,025	0,012	0,066	0,059	0,035	0,087	0,062	0,045	0,077
**Norm_Chr7**	0,012	0,028	0,010	0,034	0,032	0,016	NA	0,010	0,015	0,022	0,034	0,006	0,068	0,013	0,024	0,057	0,048	0,054	0,078	0,051	0,034	0,067
**Norm_Chr8**	0,020	0,021	0,017	0,027	0,025	0,011	0,010	NA	0,024	0,015	0,043	0,012	0,060	0,021	0,020	0,064	0,055	0,046	0,085	0,059	0,041	0,075
**Norm_Chr9**	0,005	0,042	0,015	0,046	0,046	0,030	0,014	0,023	NA	0,035	0,020	0,015	0,081	0,007	0,037	0,043	0,034	0,068	0,064	0,036	0,020	0,052
**Norm_Chr10**	0,030	0,007	0,025	0,017	0,010	0,008	0,020	0,014	0,034	NA	0,052	0,022	0,043	0,031	0,010	0,072	0,064	0,029	0,092	0,068	0,051	0,083
**Norm_Chr11**	0,022	0,063	0,033	0,065	0,069	0,050	0,033	0,041	0,021	0,056	NA	0,034	0,104	0,023	0,059	0,028	0,018	0,093	0,049	0,019	0,009	0,035
**Norm_Chr12**	0,011	0,027	0,006	0,032	0,032	0,016	0,005	0,011	0,014	0,022	0,032	NA	0,066	0,011	0,024	0,054	0,046	0,053	0,075	0,048	0,031	0,064
**Norm_Chr13**	0,064	0,035	0,062	0,032	0,033	0,045	0,055	0,048	0,069	0,038	0,086	0,058	NA	0,066	0,045	0,100	0,095	0,019	0,119	0,100	0,084	0,114
**Norm_Chr14**	0,005	0,039	0,011	0,042	0,044	0,027	0,012	0,020	0,008	0,033	0,023	0,012	0,079	NA	0,035	0,047	0,037	0,066	0,068	0,040	0,023	0,056
**Norm_Chr15**	0,027	0,011	0,023	0,018	0,013	0,011	0,018	0,015	0,030	0,009	0,047	0,021	0,043	0,028	NA	0,065	0,058	0,029	0,084	0,062	0,047	0,075
**Norm_Chr16**	0,057	0,106	0,072	0,106	0,113	0,091	0,069	0,078	0,056	0,096	0,035	0,072	0,152	0,060	0,103	NA	0,015	0,142	0,024	0,015	0,033	0,009
**Norm_Chr17**	0,040	0,085	0,054	0,086	0,091	0,071	0,052	0,060	0,039	0,076	0,020	0,054	0,127	0,042	0,082	0,013	NA	0,118	0,035	0,006	0,019	0,020
**Norm_Chr18**	0,047	0,019	0,044	0,017	0,016	0,028	0,038	0,032	0,051	0,022	0,067	0,041	0,017	0,049	0,027	0,083	0,077	NA	0,101	0,081	0,066	0,094
**Norm_Chr19**	0,084	0,136	0,102	0,134	0,144	0,120	0,097	0,105	0,084	0,125	0,062	0,101	0,183	0,088	0,135	0,025	0,040	0,176	NA	0,041	0,060	0,022
**Norm_Chr20**	0,040	0,083	0,053	0,084	0,089	0,070	0,051	0,059	0,039	0,075	0,020	0,053	0,125	0,042	0,081	0,012	0,006	0,115	0,034	NA	0,018	0,019
**Norm_Chr21**	0,023	0,065	0,035	0,066	0,070	0,052	0,034	0,042	0,022	0,057	0,009	0,035	0,107	0,024	0,062	0,028	0,018	0,095	0,050	0,018	NA	0,036
**Norm_Chr22**	0,058	0,104	0,073	0,104	0,111	0,090	0,070	0,078	0,058	0,095	0,037	0,073	0,147	0,061	0,102	0,007	0,019	0,139	0,019	0,019	0,036	NA
**Norm_all**	0,005	0,034	0,010	0,039	0,039	0,023	0,007	0,016	0,008	0,028	0,026	0,008	0,074	0,007	0,030	0,049	0,040	0,061	0,070	0,043	0,026	0,059

WES-Dual																						
	Chr1	Chr2	Chr3	Chr4	Chr5	Chr6	Chr7	Chr8	Chr9	Chr10	Chr11	Chr12	Chr13	Chr14	Chr15	Chr16	Chr17	Chr18	Chr19	Chr20	Chr21	Chr22
Norm_Chr1	NA	0,049	0,020	0,059	0,056	0,036	0,011	0,020	0,011	0,040	0,032	0,018	0,095	0,006	0,041	0,074	0,056	0,083	0,111	0,059	0,036	0,082
Norm_Chr2	0,046	NA	0,031	0,015	0,007	0,013	0,037	0,032	0,057	0,008	0,079	0,031	0,044	0,046	0,015	0,118	0,102	0,030	0,156	0,106	0,081	0,129
Norm_Chr3	0,019	0,030	NA	0,040	0,037	0,018	0,015	0,017	0,029	0,023	0,049	0,006	0,073	0,018	0,022	0,090	0,073	0,061	0,127	0,076	0,053	0,099
Norm_Chr4	0,067	0,018	0,050	NA	0,013	0,031	0,058	0,051	0,080	0,026	0,105	0,051	0,034	0,067	0,034	0,148	0,131	0,019	0,191	0,135	0,106	0,161
Norm_Chr5	0,050	0,007	0,036	0,010	NA	0,019	0,043	0,037	0,062	0,014	0,084	0,037	0,037	0,051	0,020	0,122	0,107	0,024	0,160	0,110	0,085	0,133
Norm_Chr6	0,033	0,013	0,018	0,026	0,020	NA	0,026	0,021	0,044	0,007	0,066	0,018	0,057	0,033	0,011	0,106	0,089	0,044	0,143	0,093	0,068	0,116
Norm_Chr7	0,011	0,040	0,016	0,050	0,047	0,027	NA	0,010	0,020	0,031	0,041	0,012	0,084	0,011	0,033	0,082	0,064	0,073	0,118	0,068	0,044	0,091
Norm_Chr8	0,020	0,034	0,019	0,044	0,040	0,023	0,010	NA	0,028	0,025	0,050	0,014	0,077	0,020	0,029	0,089	0,072	0,066	0,125	0,075	0,051	0,099
Norm_Chr9	0,010	0,056	0,029	0,064	0,063	0,043	0,018	0,026	NA	0,047	0,022	0,027	0,099	0,012	0,048	0,062	0,044	0,088	0,096	0,047	0,026	0,069
Norm_Chr10	0,039	0,009	0,024	0,022	0,015	0,007	0,031	0,025	0,050	NA	0,072	0,024	0,053	0,039	0,011	0,113	0,096	0,039	0,151	0,100	0,074	0,123
Norm_Chr11	0,028	0,075	0,047	0,082	0,082	0,062	0,037	0,045	0,021	0,066	NA	0,045	0,118	0,030	0,068	0,043	0,025	0,109	0,076	0,028	0,014	0,048
Norm_Chr12	0,016	0,031	0,006	0,041	0,037	0,018	0,011	0,013	0,027	0,023	0,047	NA	0,074	0,016	0,024	0,087	0,070	0,062	0,123	0,074	0,050	0,096
Norm_Chr13	0,089	0,044	0,075	0,029	0,039	0,057	0,080	0,074	0,102	0,051	0,125	0,076	NA	0,090	0,059	0,163	0,149	0,019	0,205	0,153	0,126	0,178
Norm_Chr14	0,006	0,047	0,019	0,056	0,053	0,034	0,011	0,019	0,012	0,038	0,032	0,016	0,090	NA	0,038	0,074	0,056	0,079	0,109	0,059	0,036	0,081
Norm_Chr15	0,034	0,014	0,020	0,025	0,018	0,010	0,028	0,025	0,044	0,010	0,065	0,022	0,052	0,034	NA	0,103	0,087	0,040	0,138	0,091	0,068	0,112
Norm_Chr16	0,073	0,126	0,096	0,129	0,134	0,112	0,083	0,090	0,067	0,114	0,048	0,094	0,172	0,077	0,121	NA	0,022	0,165	0,032	0,021	0,042	0,010
Norm_Chr17	0,051	0,100	0,072	0,104	0,107	0,087	0,059	0,067	0,044	0,089	0,026	0,070	0,143	0,054	0,094	0,020	NA	0,135	0,051	0,007	0,021	0,025
Norm_Chr18	0,070	0,028	0,056	0,014	0,022	0,040	0,062	0,056	0,082	0,034	0,103	0,057	0,017	0,070	0,040	0,140	0,126	NA	0,178	0,130	0,104	0,152
Norm_Chr19	0,100	0,153	0,124	0,153	0,161	0,139	0,110	0,117	0,096	0,141	0,078	0,122	0,197	0,105	0,149	0,030	0,052	0,192	NA	0,051	0,070	0,030
Norm_Chr20	0,051	0,100	0,072	0,104	0,107	0,087	0,060	0,067	0,045	0,089	0,027	0,070	0,142	0,054	0,094	0,018	0,007	0,135	0,049	NA	0,021	0,023
Norm_Chr21	0,033	0,081	0,054	0,087	0,089	0,069	0,041	0,049	0,027	0,071	0,015	0,051	0,125	0,036	0,075	0,039	0,022	0,116	0,072	0,023	NA	0,046
Norm_Chr22	0,072	0,122	0,093	0,124	0,129	0,109	0,081	0,088	0,066	0,111	0,048	0,092	0,165	0,075	0,116	0,008	0,024	0,158	0,029	0,023	0,043	NA
Norm_all	0,005	0,046	0,019	0,055	0,052	0,033	0,008	0,016	0,012	0,037	0,032	0,016	0,089	0,006	0,038	0,072	0,055	0,078	0,107	0,058	0,035	0,080
