# Supplementary material for: Identification of autosomal and sex chromosome aneuploidies using next generation sequencing
Source: Bioinformatics. 2026 Mar 16;42(3):btag104. doi: 10.1093/bioinformatics/btag104 (PMC13032822; doi:10.1093/bioinformatics/btag104)
Supplement: btag104_Supplementary_Data [file btag104_supplementary_data.zip › SuppTable_1.docx]

|  | Controls | | | HSJD cohort | | |
| --- | --- | --- | --- | --- | --- | --- |
|  | **CES** | **WES-Single** | **WES-Dual** | **CES** | **WES-Single** | **WES-Dual** |
| Mean | 146.77 | 69.49 | 115.33 | 141.79 | 74.49 | 116.33 |
| Median | 147.20 | 64.86 | 107.70 | 141.66 | 70.73 | 107.89 |
| 1st quartile | 133.36 | 56.18 | 97.62 | 127.98 | 60.43 | 98.15 |
| 3rd quartile | 161.01 | 77.11 | 119.14 | 154.80 | 78.54 | 119.45 |
