# Supplementary material for: Identification of autosomal and sex chromosome aneuploidies using next generation sequencing
Source: Bioinformatics. 2026 Mar 16;42(3):btag104. doi: 10.1093/bioinformatics/btag104 (PMC13032822; doi:10.1093/bioinformatics/btag104)
Supplement: btag104_Supplementary_Data [file btag104_supplementary_data.zip › SuppTable_2.docx]

| **Coverage** | **Z-score** |
| --- | --- |
| 10 | -4.737 |
| 20 | -4.391 |
| 30 | -4.044 |
| 40 | -3.698 |
| 50 | -3.352 |
| 60 | -3.005 |
| 70 | -2.659 |
| 80 | -2.313 |
| 90 | -1.966 |
| 100 | -1.620 |
