# Supplementary material for: Identification of autosomal and sex chromosome aneuploidies using next generation sequencing
Source: Bioinformatics. 2026 Mar 16;42(3):btag104. doi: 10.1093/bioinformatics/btag104 (PMC13032822; doi:10.1093/bioinformatics/btag104)
Supplement: btag104_Supplementary_Data [file btag104_supplementary_data.zip › SuppTable_3.docx]

| Chromosome | CES Vs WES (p-value) | WES-Single Vs WES-Dual (p-value) |
| --- | --- | --- |
| Chr1 | 0.00E+00 | 2.08E-01 |
| Chr2 | 0.00E+00 | 2.18E-71 |
| Chr3 | 5.88E-90 | 5.30E-93 |
| Chr4 | 0.00E+00 | 1.18E-75 |
| Chr5 | 4.10E-08 | 4.03E-76 |
| Chr6 | 4.74E-17 | 2.05E-66 |
| Chr7 | 3.41E-312 | 1.63E-62 |
| Chr8 | 0.00E+00 | 5.33E-23 |
| Chr9 | 0.00E+00 | 4.65E-14 |
| Chr10 | 3.39E-175 | 6.29E-69 |
| Chr11 | 2.12E-33 | 1.46E-58 |
| Chr12 | 2.72E-215 | 1.14E-62 |
| Chr13 | 1.26E-61 | 1.03E-54 |
| Chr14 | 0.00E+00 | 5.62E-19 |
| Chr15 | 1.28E-53 | 1.83E-87 |
| Chr16 | 9.53E-87 | 4.79E-76 |
| Chr17 | 8.43E-207 | 4.19E-82 |
| Chr18 | 1.28E-212 | 2.03E-73 |
| Chr19 | 0.00E+00 | 3.86E-83 |
| Chr20 | 0.00E+00 | 8.35E-70 |
| Chr21 | 0.00E+00 | 3.23E-18 |
| Chr22 | 3.94E-275 | 2.30E-66 |
