# Supplementary material for: Identification of autosomal and sex chromosome aneuploidies using next generation sequencing
Source: Bioinformatics. 2026 Mar 16;42(3):btag104. doi: 10.1093/bioinformatics/btag104 (PMC13032822; doi:10.1093/bioinformatics/btag104)
Supplement: btag104_Supplementary_Data [file btag104_supplementary_data.zip › SuppTable_4.docx]

| **CES** | | | | | | | | | | | | | | | | | | | | | | |
| --- | --- | --- | --- | --- | --- | --- | --- | --- | --- | --- | --- | --- | --- | --- | --- | --- | --- | --- | --- | --- | --- | --- |
|  | **Chr1** | **Chr2** | **Chr3** | **Chr4** | **Chr5** | **Chr6** | **Chr7** | **Chr8** | **Chr9** | **Chr10** | **Chr11** | **Chr12** | **Chr13** | **Chr14** | **Chr15** | **Chr16** | **Chr17** | **Chr18** | **Chr19** | **Chr20** | **Chr21** | **Chr22** |
| **Norm_Chr1** | NA | 0,040 | 0,012 | 0,055 | 0,044 | 0,036 | 0,033 | 0,030 | 0,014 | 0,031 | 0,017 | 0,014 | 0,069 | 0,013 | 0,022 | 0,046 | 0,036 | 0,041 | 0,073 | 0,032 | 0,034 | 0,055 |
| **Norm_Chr2** | 0,034 | NA | 0,023 | 0,018 | 0,012 | 0,009 | 0,009 | 0,016 | 0,041 | 0,013 | 0,047 | 0,021 | 0,031 | 0,035 | 0,015 | 0,072 | 0,061 | 0,013 | 0,091 | 0,057 | 0,056 | 0,079 |
| **Norm_Chr3** | 0,013 | 0,030 | NA | 0,047 | 0,034 | 0,027 | 0,023 | 0,021 | 0,022 | 0,021 | 0,028 | 0,005 | 0,061 | 0,017 | 0,012 | 0,058 | 0,046 | 0,033 | 0,084 | 0,042 | 0,043 | 0,067 |
| **Norm_Chr4** | 0,054 | 0,021 | 0,042 | NA | 0,015 | 0,019 | 0,023 | 0,032 | 0,061 | 0,027 | 0,069 | 0,040 | 0,020 | 0,056 | 0,034 | 0,094 | 0,083 | 0,015 | 0,112 | 0,077 | 0,075 | 0,102 |
| **Norm_Chr5** | 0,047 | 0,015 | 0,033 | 0,017 | NA | 0,009 | 0,012 | 0,020 | 0,053 | 0,015 | 0,061 | 0,031 | 0,026 | 0,047 | 0,024 | 0,087 | 0,075 | 0,013 | 0,107 | 0,070 | 0,068 | 0,095 |
| **Norm_Chr6** | 0,040 | 0,011 | 0,027 | 0,021 | 0,009 | NA | 0,008 | 0,015 | 0,047 | 0,010 | 0,054 | 0,025 | 0,032 | 0,039 | 0,018 | 0,081 | 0,069 | 0,013 | 0,102 | 0,064 | 0,063 | 0,090 |
| **Norm_Chr7** | 0,036 | 0,012 | 0,023 | 0,025 | 0,012 | 0,008 | NA | 0,010 | 0,043 | 0,007 | 0,050 | 0,021 | 0,037 | 0,035 | 0,014 | 0,077 | 0,064 | 0,017 | 0,099 | 0,060 | 0,059 | 0,085 |
| **Norm_Chr8** | 0,031 | 0,019 | 0,019 | 0,032 | 0,019 | 0,014 | 0,009 | NA | 0,037 | 0,010 | 0,043 | 0,019 | 0,041 | 0,028 | 0,013 | 0,068 | 0,056 | 0,024 | 0,090 | 0,053 | 0,051 | 0,077 |
| **Norm_Chr9** | 0,016 | 0,057 | 0,024 | 0,072 | 0,059 | 0,051 | 0,047 | 0,043 | NA | 0,044 | 0,012 | 0,026 | 0,087 | 0,021 | 0,037 | 0,039 | 0,030 | 0,055 | 0,071 | 0,024 | 0,027 | 0,050 |
| **Norm_Chr10** | 0,033 | 0,016 | 0,020 | 0,028 | 0,015 | 0,009 | 0,007 | 0,011 | 0,039 | NA | 0,047 | 0,018 | 0,039 | 0,032 | 0,012 | 0,073 | 0,061 | 0,017 | 0,095 | 0,057 | 0,056 | 0,081 |
| **Norm_Chr11** | 0,018 | 0,059 | 0,028 | 0,074 | 0,061 | 0,054 | 0,049 | 0,045 | 0,011 | 0,048 | NA | 0,030 | 0,088 | 0,021 | 0,040 | 0,033 | 0,023 | 0,059 | 0,064 | 0,019 | 0,023 | 0,042 |
| **Norm_Chr12** | 0,015 | 0,028 | 0,005 | 0,045 | 0,033 | 0,026 | 0,022 | 0,020 | 0,024 | 0,019 | 0,031 | NA | 0,059 | 0,019 | 0,011 | 0,060 | 0,049 | 0,031 | 0,087 | 0,044 | 0,045 | 0,069 |
| **Norm_Chr13** | 0,069 | 0,037 | 0,055 | 0,020 | 0,024 | 0,030 | 0,034 | 0,041 | 0,073 | 0,037 | 0,081 | 0,053 | NA | 0,068 | 0,046 | 0,104 | 0,093 | 0,029 | 0,119 | 0,087 | 0,083 | 0,112 |
| **Norm_Chr14** | 0,013 | 0,039 | 0,015 | 0,053 | 0,042 | 0,035 | 0,031 | 0,027 | 0,017 | 0,030 | 0,019 | 0,016 | 0,065 | NA | 0,023 | 0,046 | 0,035 | 0,041 | 0,071 | 0,031 | 0,033 | 0,055 |
| **Norm_Chr15** | 0,023 | 0,018 | 0,011 | 0,035 | 0,023 | 0,017 | 0,013 | 0,013 | 0,032 | 0,011 | 0,038 | 0,010 | 0,048 | 0,024 | NA | 0,065 | 0,053 | 0,023 | 0,088 | 0,049 | 0,049 | 0,073 |
| **Norm_Chr16** | 0,061 | 0,110 | 0,068 | 0,123 | 0,106 | 0,097 | 0,092 | 0,088 | 0,044 | 0,091 | 0,040 | 0,070 | 0,137 | 0,062 | 0,083 | NA | 0,019 | 0,102 | 0,042 | 0,017 | 0,016 | 0,018 |
| **Norm_Chr17** | 0,045 | 0,090 | 0,052 | 0,105 | 0,087 | 0,080 | 0,075 | 0,070 | 0,032 | 0,073 | 0,027 | 0,054 | 0,117 | 0,046 | 0,065 | 0,018 | NA | 0,086 | 0,051 | 0,011 | 0,012 | 0,026 |
| **Norm_Chr18** | 0,047 | 0,018 | 0,034 | 0,017 | 0,014 | 0,014 | 0,018 | 0,027 | 0,053 | 0,019 | 0,063 | 0,031 | 0,033 | 0,049 | 0,026 | 0,090 | 0,078 | NA | 0,111 | 0,072 | 0,071 | 0,099 |
| **Norm_Chr19** | 0,157 | 0,228 | 0,163 | 0,241 | 0,212 | 0,202 | 0,194 | 0,190 | 0,130 | 0,194 | 0,127 | 0,165 | 0,256 | 0,159 | 0,185 | 0,070 | 0,087 | 0,209 | NA | 0,088 | 0,078 | 0,055 |
| **Norm_Chr20** | 0,043 | 0,092 | 0,052 | 0,106 | 0,089 | 0,081 | 0,076 | 0,071 | 0,028 | 0,074 | 0,024 | 0,054 | 0,120 | 0,045 | 0,066 | 0,018 | 0,012 | 0,086 | 0,056 | NA | 0,011 | 0,030 |
| **Norm_Chr21** | 0,055 | 0,105 | 0,062 | 0,120 | 0,101 | 0,092 | 0,086 | 0,081 | 0,036 | 0,084 | 0,034 | 0,064 | 0,134 | 0,054 | 0,077 | 0,019 | 0,015 | 0,098 | 0,057 | 0,012 | NA | 0,032 |
| **Norm_Chr22** | 0,075 | 0,127 | 0,082 | 0,140 | 0,121 | 0,113 | 0,107 | 0,104 | 0,058 | 0,106 | 0,054 | 0,084 | 0,154 | 0,078 | 0,098 | 0,018 | 0,029 | 0,118 | 0,035 | 0,030 | 0,027 | NA |
| **Norm_all** | 0,012 | 0,034 | 0,006 | 0,050 | 0,037 | 0,030 | 0,025 | 0,021 | 0,020 | 0,023 | 0,025 | 0,009 | 0,063 | 0,012 | 0,016 | 0,054 | 0,042 | 0,036 | 0,080 | 0,038 | 0,039 | 0,063 |
|  | | | | | | | | | | | | | | | | | | | | | | |
| **WES-Single** | | | | | | | | | | | | | | | | | | | | | | |
|  | **Chr1** | **Chr2** | **Chr3** | **Chr4** | **Chr5** | **Chr6** | **Chr7** | **Chr8** | **Chr9** | **Chr10** | **Chr11** | **Chr12** | **Chr13** | **Chr14** | **Chr15** | **Chr16** | **Chr17** | **Chr18** | **Chr19** | **Chr20** | **Chr21** | **Chr22** |
| **Norm_Chr1** | NA | 0,040 | 0,012 | 0,044 | 0,045 | 0,028 | 0,012 | 0,021 | 0,005 | 0,034 | 0,023 | 0,012 | 0,082 | 0,006 | 0,036 | 0,048 | 0,038 | 0,068 | 0,070 | 0,041 | 0,024 | 0,057 |
| **Norm_Chr2** | 0,033 | NA | 0,029 | 0,011 | 0,004 | 0,011 | 0,024 | 0,018 | 0,038 | 0,007 | 0,055 | 0,026 | 0,038 | 0,035 | 0,012 | 0,074 | 0,067 | 0,023 | 0,094 | 0,071 | 0,054 | 0,085 |
| **Norm_Chr3** | 0,010 | 0,030 | NA | 0,033 | 0,034 | 0,019 | 0,009 | 0,015 | 0,014 | 0,025 | 0,030 | 0,006 | 0,069 | 0,010 | 0,026 | 0,053 | 0,045 | 0,055 | 0,073 | 0,047 | 0,030 | 0,062 |
| **Norm_Chr4** | 0,043 | 0,013 | 0,036 | NA | 0,013 | 0,020 | 0,033 | 0,027 | 0,048 | 0,018 | 0,066 | 0,035 | 0,040 | 0,044 | 0,023 | 0,087 | 0,079 | 0,024 | 0,108 | 0,083 | 0,065 | 0,099 |
| **Norm_Chr5** | 0,035 | 0,004 | 0,031 | 0,011 | NA | 0,014 | 0,026 | 0,020 | 0,040 | 0,009 | 0,057 | 0,028 | 0,033 | 0,037 | 0,014 | 0,075 | 0,068 | 0,018 | 0,094 | 0,071 | 0,056 | 0,086 |
| **Norm_Chr6** | 0,024 | 0,012 | 0,018 | 0,018 | 0,016 | NA | 0,014 | 0,009 | 0,028 | 0,008 | 0,046 | 0,015 | 0,050 | 0,025 | 0,012 | 0,066 | 0,059 | 0,035 | 0,087 | 0,062 | 0,045 | 0,077 |
| **Norm_Chr7** | 0,012 | 0,028 | 0,010 | 0,034 | 0,032 | 0,016 | NA | 0,010 | 0,015 | 0,022 | 0,034 | 0,006 | 0,068 | 0,013 | 0,024 | 0,057 | 0,048 | 0,054 | 0,078 | 0,051 | 0,034 | 0,067 |
| **Norm_Chr8** | 0,020 | 0,021 | 0,017 | 0,027 | 0,025 | 0,011 | 0,010 | NA | 0,024 | 0,015 | 0,043 | 0,012 | 0,060 | 0,021 | 0,020 | 0,064 | 0,055 | 0,046 | 0,085 | 0,059 | 0,041 | 0,075 |
| **Norm_Chr9** | 0,005 | 0,042 | 0,015 | 0,046 | 0,046 | 0,030 | 0,014 | 0,023 | NA | 0,035 | 0,020 | 0,015 | 0,081 | 0,007 | 0,037 | 0,043 | 0,034 | 0,068 | 0,064 | 0,036 | 0,020 | 0,052 |
| **Norm_Chr10** | 0,030 | 0,007 | 0,025 | 0,017 | 0,010 | 0,008 | 0,020 | 0,014 | 0,034 | NA | 0,052 | 0,022 | 0,043 | 0,031 | 0,010 | 0,072 | 0,064 | 0,029 | 0,092 | 0,068 | 0,051 | 0,083 |
| **Norm_Chr11** | 0,022 | 0,063 | 0,033 | 0,065 | 0,069 | 0,050 | 0,033 | 0,041 | 0,021 | 0,056 | NA | 0,034 | 0,104 | 0,023 | 0,059 | 0,028 | 0,018 | 0,093 | 0,049 | 0,019 | 0,009 | 0,035 |
| **Norm_Chr12** | 0,011 | 0,027 | 0,006 | 0,032 | 0,032 | 0,016 | 0,005 | 0,011 | 0,014 | 0,022 | 0,032 | NA | 0,066 | 0,011 | 0,024 | 0,054 | 0,046 | 0,053 | 0,075 | 0,048 | 0,031 | 0,064 |
| **Norm_Chr13** | 0,064 | 0,035 | 0,062 | 0,032 | 0,033 | 0,045 | 0,055 | 0,048 | 0,069 | 0,038 | 0,086 | 0,058 | NA | 0,066 | 0,045 | 0,100 | 0,095 | 0,019 | 0,119 | 0,100 | 0,084 | 0,114 |
| **Norm_Chr14** | 0,005 | 0,039 | 0,011 | 0,042 | 0,044 | 0,027 | 0,012 | 0,020 | 0,008 | 0,033 | 0,023 | 0,012 | 0,079 | NA | 0,035 | 0,047 | 0,037 | 0,066 | 0,068 | 0,040 | 0,023 | 0,056 |
| **Norm_Chr15** | 0,027 | 0,011 | 0,023 | 0,018 | 0,013 | 0,011 | 0,018 | 0,015 | 0,030 | 0,009 | 0,047 | 0,021 | 0,043 | 0,028 | NA | 0,065 | 0,058 | 0,029 | 0,084 | 0,062 | 0,047 | 0,075 |
| **Norm_Chr16** | 0,057 | 0,106 | 0,072 | 0,106 | 0,113 | 0,091 | 0,069 | 0,078 | 0,056 | 0,096 | 0,035 | 0,072 | 0,152 | 0,060 | 0,103 | NA | 0,015 | 0,142 | 0,024 | 0,015 | 0,033 | 0,009 |
| **Norm_Chr17** | 0,040 | 0,085 | 0,054 | 0,086 | 0,091 | 0,071 | 0,052 | 0,060 | 0,039 | 0,076 | 0,020 | 0,054 | 0,127 | 0,042 | 0,082 | 0,013 | NA | 0,118 | 0,035 | 0,006 | 0,019 | 0,020 |
| **Norm_Chr18** | 0,047 | 0,019 | 0,044 | 0,017 | 0,016 | 0,028 | 0,038 | 0,032 | 0,051 | 0,022 | 0,067 | 0,041 | 0,017 | 0,049 | 0,027 | 0,083 | 0,077 | NA | 0,101 | 0,081 | 0,066 | 0,094 |
| **Norm_Chr19** | 0,084 | 0,136 | 0,102 | 0,134 | 0,144 | 0,120 | 0,097 | 0,105 | 0,084 | 0,125 | 0,062 | 0,101 | 0,183 | 0,088 | 0,135 | 0,025 | 0,040 | 0,176 | NA | 0,041 | 0,060 | 0,022 |
| **Norm_Chr20** | 0,040 | 0,083 | 0,053 | 0,084 | 0,089 | 0,070 | 0,051 | 0,059 | 0,039 | 0,075 | 0,020 | 0,053 | 0,125 | 0,042 | 0,081 | 0,012 | 0,006 | 0,115 | 0,034 | NA | 0,018 | 0,019 |
| **Norm_Chr21** | 0,023 | 0,065 | 0,035 | 0,066 | 0,070 | 0,052 | 0,034 | 0,042 | 0,022 | 0,057 | 0,009 | 0,035 | 0,107 | 0,024 | 0,062 | 0,028 | 0,018 | 0,095 | 0,050 | 0,018 | NA | 0,036 |
| **Norm_Chr22** | 0,058 | 0,104 | 0,073 | 0,104 | 0,111 | 0,090 | 0,070 | 0,078 | 0,058 | 0,095 | 0,037 | 0,073 | 0,147 | 0,061 | 0,102 | 0,007 | 0,019 | 0,139 | 0,019 | 0,019 | 0,036 | NA |
| **Norm_all** | 0,005 | 0,034 | 0,010 | 0,039 | 0,039 | 0,023 | 0,007 | 0,016 | 0,008 | 0,028 | 0,026 | 0,008 | 0,074 | 0,007 | 0,030 | 0,049 | 0,040 | 0,061 | 0,070 | 0,043 | 0,026 | 0,059 |
|  | | | | | | | | | | | | | | | | | | | | | | |
| **WES-Dual** | | | | | | | | | | | | | | | | | | | | | | |
|  | **Chr1** | **Chr2** | **Chr3** | **Chr4** | **Chr5** | **Chr6** | **Chr7** | **Chr8** | **Chr9** | **Chr10** | **Chr11** | **Chr12** | **Chr13** | **Chr14** | **Chr15** | **Chr16** | **Chr17** | **Chr18** | **Chr19** | **Chr20** | **Chr21** | **Chr22** |
| **Norm_Chr1** | NA | 0,049 | 0,020 | 0,059 | 0,056 | 0,036 | 0,011 | 0,020 | 0,011 | 0,040 | 0,032 | 0,018 | 0,095 | 0,006 | 0,041 | 0,074 | 0,056 | 0,083 | 0,111 | 0,059 | 0,036 | 0,082 |
| **Norm_Chr2** | 0,046 | NA | 0,031 | 0,015 | 0,007 | 0,013 | 0,037 | 0,032 | 0,057 | 0,008 | 0,079 | 0,031 | 0,044 | 0,046 | 0,015 | 0,118 | 0,102 | 0,030 | 0,156 | 0,106 | 0,081 | 0,129 |
| **Norm_Chr3** | 0,019 | 0,030 | NA | 0,040 | 0,037 | 0,018 | 0,015 | 0,017 | 0,029 | 0,023 | 0,049 | 0,006 | 0,073 | 0,018 | 0,022 | 0,090 | 0,073 | 0,061 | 0,127 | 0,076 | 0,053 | 0,099 |
| **Norm_Chr4** | 0,067 | 0,018 | 0,050 | NA | 0,013 | 0,031 | 0,058 | 0,051 | 0,080 | 0,026 | 0,105 | 0,051 | 0,034 | 0,067 | 0,034 | 0,148 | 0,131 | 0,019 | 0,191 | 0,135 | 0,106 | 0,161 |
| **Norm_Chr5** | 0,050 | 0,007 | 0,036 | 0,010 | NA | 0,019 | 0,043 | 0,037 | 0,062 | 0,014 | 0,084 | 0,037 | 0,037 | 0,051 | 0,020 | 0,122 | 0,107 | 0,024 | 0,160 | 0,110 | 0,085 | 0,133 |
| **Norm_Chr6** | 0,033 | 0,013 | 0,018 | 0,026 | 0,020 | NA | 0,026 | 0,021 | 0,044 | 0,007 | 0,066 | 0,018 | 0,057 | 0,033 | 0,011 | 0,106 | 0,089 | 0,044 | 0,143 | 0,093 | 0,068 | 0,116 |
| **Norm_Chr7** | 0,011 | 0,040 | 0,016 | 0,050 | 0,047 | 0,027 | NA | 0,010 | 0,020 | 0,031 | 0,041 | 0,012 | 0,084 | 0,011 | 0,033 | 0,082 | 0,064 | 0,073 | 0,118 | 0,068 | 0,044 | 0,091 |
| **Norm_Chr8** | 0,020 | 0,034 | 0,019 | 0,044 | 0,040 | 0,023 | 0,010 | NA | 0,028 | 0,025 | 0,050 | 0,014 | 0,077 | 0,020 | 0,029 | 0,089 | 0,072 | 0,066 | 0,125 | 0,075 | 0,051 | 0,099 |
| **Norm_Chr9** | 0,010 | 0,056 | 0,029 | 0,064 | 0,063 | 0,043 | 0,018 | 0,026 | NA | 0,047 | 0,022 | 0,027 | 0,099 | 0,012 | 0,048 | 0,062 | 0,044 | 0,088 | 0,096 | 0,047 | 0,026 | 0,069 |
| **Norm_Chr10** | 0,039 | 0,009 | 0,024 | 0,022 | 0,015 | 0,007 | 0,031 | 0,025 | 0,050 | NA | 0,072 | 0,024 | 0,053 | 0,039 | 0,011 | 0,113 | 0,096 | 0,039 | 0,151 | 0,100 | 0,074 | 0,123 |
| **Norm_Chr11** | 0,028 | 0,075 | 0,047 | 0,082 | 0,082 | 0,062 | 0,037 | 0,045 | 0,021 | 0,066 | NA | 0,045 | 0,118 | 0,030 | 0,068 | 0,043 | 0,025 | 0,109 | 0,076 | 0,028 | 0,014 | 0,048 |
| **Norm_Chr12** | 0,016 | 0,031 | 0,006 | 0,041 | 0,037 | 0,018 | 0,011 | 0,013 | 0,027 | 0,023 | 0,047 | NA | 0,074 | 0,016 | 0,024 | 0,087 | 0,070 | 0,062 | 0,123 | 0,074 | 0,050 | 0,096 |
| **Norm_Chr13** | 0,089 | 0,044 | 0,075 | 0,029 | 0,039 | 0,057 | 0,080 | 0,074 | 0,102 | 0,051 | 0,125 | 0,076 | NA | 0,090 | 0,059 | 0,163 | 0,149 | 0,019 | 0,205 | 0,153 | 0,126 | 0,178 |
| **Norm_Chr14** | 0,006 | 0,047 | 0,019 | 0,056 | 0,053 | 0,034 | 0,011 | 0,019 | 0,012 | 0,038 | 0,032 | 0,016 | 0,090 | NA | 0,038 | 0,074 | 0,056 | 0,079 | 0,109 | 0,059 | 0,036 | 0,081 |
| **Norm_Chr15** | 0,034 | 0,014 | 0,020 | 0,025 | 0,018 | 0,010 | 0,028 | 0,025 | 0,044 | 0,010 | 0,065 | 0,022 | 0,052 | 0,034 | NA | 0,103 | 0,087 | 0,040 | 0,138 | 0,091 | 0,068 | 0,112 |
| **Norm_Chr16** | 0,073 | 0,126 | 0,096 | 0,129 | 0,134 | 0,112 | 0,083 | 0,090 | 0,067 | 0,114 | 0,048 | 0,094 | 0,172 | 0,077 | 0,121 | NA | 0,022 | 0,165 | 0,032 | 0,021 | 0,042 | 0,010 |
| **Norm_Chr17** | 0,051 | 0,100 | 0,072 | 0,104 | 0,107 | 0,087 | 0,059 | 0,067 | 0,044 | 0,089 | 0,026 | 0,070 | 0,143 | 0,054 | 0,094 | 0,020 | NA | 0,135 | 0,051 | 0,007 | 0,021 | 0,025 |
| **Norm_Chr18** | 0,070 | 0,028 | 0,056 | 0,014 | 0,022 | 0,040 | 0,062 | 0,056 | 0,082 | 0,034 | 0,103 | 0,057 | 0,017 | 0,070 | 0,040 | 0,140 | 0,126 | NA | 0,178 | 0,130 | 0,104 | 0,152 |
| **Norm_Chr19** | 0,100 | 0,153 | 0,124 | 0,153 | 0,161 | 0,139 | 0,110 | 0,117 | 0,096 | 0,141 | 0,078 | 0,122 | 0,197 | 0,105 | 0,149 | 0,030 | 0,052 | 0,192 | NA | 0,051 | 0,070 | 0,030 |
| **Norm_Chr20** | 0,051 | 0,100 | 0,072 | 0,104 | 0,107 | 0,087 | 0,060 | 0,067 | 0,045 | 0,089 | 0,027 | 0,070 | 0,142 | 0,054 | 0,094 | 0,018 | 0,007 | 0,135 | 0,049 | NA | 0,021 | 0,023 |
| **Norm_Chr21** | 0,033 | 0,081 | 0,054 | 0,087 | 0,089 | 0,069 | 0,041 | 0,049 | 0,027 | 0,071 | 0,015 | 0,051 | 0,125 | 0,036 | 0,075 | 0,039 | 0,022 | 0,116 | 0,072 | 0,023 | NA | 0,046 |
| **Norm_Chr22** | 0,072 | 0,122 | 0,093 | 0,124 | 0,129 | 0,109 | 0,081 | 0,088 | 0,066 | 0,111 | 0,048 | 0,092 | 0,165 | 0,075 | 0,116 | 0,008 | 0,024 | 0,158 | 0,029 | 0,023 | 0,043 | NA |
| **Norm_all** | 0,005 | 0,046 | 0,019 | 0,055 | 0,052 | 0,033 | 0,008 | 0,016 | 0,012 | 0,037 | 0,032 | 0,016 | 0,089 | 0,006 | 0,038 | 0,072 | 0,055 | 0,078 | 0,107 | 0,058 | 0,035 | 0,080 |
